# Vascular Injury in the Zebrafish Tail Modulates Blood Flow and Peak Wall Shear Stress to Restore Embryonic Circular Network

**DOI:** 10.3389/fcvm.2022.841101

**Published:** 2022-03-18

**Authors:** Kyung In Baek, Shyr-Shea Chang, Chih-Chiang Chang, Mehrdad Roustaei, Yichen Ding, Yixuan Wang, Justin Chen, Ryan O'Donnell, Hong Chen, Julianne W. Ashby, Xiaolei Xu, Julia J. Mack, Susana Cavallero, Marcus Roper, Tzung K. Hsiai

**Affiliations:** ^1^Department of Medicine and Bioengineering, University of California, Los Angeles, Los Angeles, CA, United States; ^2^Department of Mathematics, University of California, Los Angeles, Los Angeles, CA, United States; ^3^Center for Studies in Physics and Biology, The Rockefeller University, New York, NY, United States; ^4^Developmental Biology Program, Sloan Kettering Institute, New York, NY, United States; ^5^Vascular Biology Program, Boston Children's Hospital, Harvard Medical School, Boston, MA, United States; ^6^Division of Cardiology, Department of Medicine, School of Medicine, University of California, Los Angeles, Los Angeles, CA, United States; ^7^Zebrafish Genetics, Mayo Clinic, Rochester, MN, United States; ^8^Veterans Affairs Greater Los Angeles Healthcare System, Los Angeles, CA, United States

**Keywords:** biophysics, peak wall shear stress, Notch-ephrinb2 signaling, vascular loop formation, vascular injury and repair

## Abstract

Mechano-responsive signaling pathways enable blood vessels within a connected network to structurally adapt to partition of blood flow between organ systems. Wall shear stress (WSS) modulates endothelial cell proliferation and arteriovenous specification. Here, we study vascular regeneration in a zebrafish model by using tail amputation to disrupt the embryonic circulatory loop (ECL) at 3 days post fertilization (dpf). We observed a local increase in blood flow and peak WSS in the Segmental Artery (SeA) immediately adjacent to the amputation site. By manipulating blood flow and WSS via changes in blood viscosity and myocardial contractility, we show that the angiogenic Notch-ephrinb2 cascade is hemodynamically activated in the SeA to guide arteriogenesis and network reconnection. Taken together, ECL amputation induces changes in microvascular topology to partition blood flow and increase WSS-mediated Notch-ephrinb2 pathway, promoting new vascular arterial loop formation and restoring microcirculation.

## Graphical Abstract

The proposed mechanism of injury-mediated Notch and vascular loop formation. 
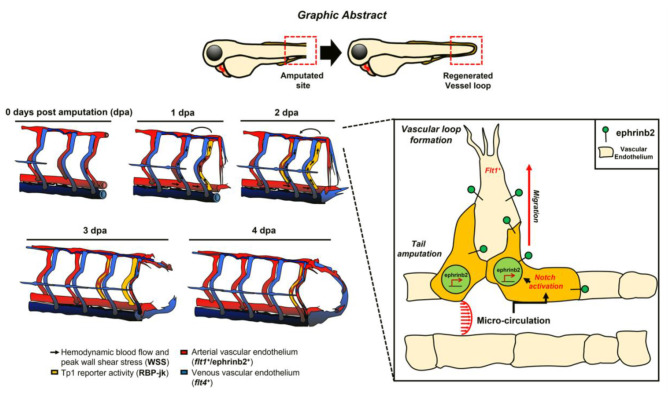

## Introduction

Microvascular networks achieve complex feats of traffic control: perfusing tissues throughout the body, avoiding hydraulic short circuits, and dynamically reallocating fluxes in response to tissue demands. Microvascular dysfunction is known to associate with several cardiometabolic disorders including diabetes, hypertension, hyperlipidemia, and obesity (1, 2). Mechano-responsive signaling pathways enable vessels within a connected network to structurally adapt in order to properly partition blood flow between different organ systems. The endothelium, the inner lining of vessel walls, transduces biomechanical wall shear stress (WSS) from blood viscosity and flow (3, 4). In the WSS set-point model, blood vessel growth is responsive to WSS as vascular angiogenesis is programmed to produce an increase in endoluminal radius in response to high WSS or vice versa (5, 6). WSS is known to shape the formation of microvascular networks, allowing superfluous vessels to be pruned (7) and promoting angiogenesis at sites of high flow (8). Since injured vessels carry no flow in the absence of WSS, the biomechanical mechanisms underlying vascular injury-mediated changes in hemodynamics that facilitate vascular network regeneration remains an unexplored question.

Endothelial Notch is a well-recognized mechano-sensitive signaling pathway (9). In response to hemodynamic shear forces, proteolytic activation of Notch receptors (Notch1-4) releases Notch Intracellular Cytoplasmic Domain (NICD) that transmigrates to the nucleus (10) to increase the transcription of target proteins including Hairy and enhancer of split-1 (Hes1) and ephrinb2 (11). During cardiac morphogenesis, WSS induces Notch1 and the subsequent activation of ephrinb2-neuregulin 1/erb-b2 receptor tyrosine kinase 2 signaling pathway to initiate cardiac trabeculation (12, 13). In the arterial circulation, pulsatile WSS in the relative straight segments and oscillatory WSS in the branching points differentially regulate Delta/Serrate/Lag ligands in vascular endothelial and smooth muscle cells (14).

The transmembrane ephrinb2 ligand mediates developmental angiogenesis and arteriovenous specifications. Targeted disruption in ephrinb2 expression induces arteriovenous malformation and defective sprout formation. In addition, homozygous knockout of ephrinb2 arrests primitive arterial and venous vessel differentiation and proliferation (15–17). Cell-cell contact mediates Notch-ephrinb2 bidirectional signaling to EphB4 receptor tyrosine kinase (18, 19). Reciprocal expression of ephrinb2/EphB4 primes the dorsal aorta (DA) and posterior cardinal vein (PCV) to undergo arteriovenous specification in zebrafish embryos (20, 21).

Embryonic zebrafish (*Danio rerio*) emerged as a crucial developmental model due to conserved genetics during organogenesis (22, 23). Combined with the latest genomic engineering techniques, its unique optical transparency allows longitudinal analyses in developing cardiovascular system (13, 24). In addition, simple husbandry, ease of genetic manipulation, and impressive regenerative capacity of zebrafish allows a large-scale pharmacogenetic screening with therapeutic potential (25). In the zebrafish microvascular network, the DA carries blood to a parallel network of segmental arteries (SeA) that connect to the dorsal longitudinal anastomotic vessel (DLAV). Segmental veins (SeV) carry the venous blood from the DLAV to the principal cardinal vein (PCV), and the DA connects with the PCV to form an embryonic circulatory loop (ECL) in the tail region. Using the zebrafish model of tail amputation (26–28), we sought to determine how local hemodynamic WSS cues drive vascular regeneration following injury. Tail amputation resulted in severing the ECL, creating a direct anastomotic connection between artery and vein to focus flow into a single segmental (Se) vessel, along with a local increase in peak WSS. This increase in WSS is accompanied by elevated Notch activity. Genetic and pharmacologic manipulations to elevate WSS in the SeA recapitulated the induction of Notch signaling to guide formation of a new DLAV-PCV loop. Our results reveal that WSS-mediated Notch signaling reconstitutes the arterial network by increasing endothelial ephrinb2 expression, leading to differentiation of new vessels to restore microcirculation.

## Materials and Methods

### Study Approval

Zebrafish experiments were performed in compliance with the Institutional Animal Care and Use Committees (IACUC) at the University of California, Los Angeles (UCLA) under animal welfare assurance number A3196-01.

### The Transgenic Zebrafish Tail Amputation Model for Vascular Injury and Regeneration

Zebrafish embryos were harvested from natural mating at the UCLA Zebrafish Core Facility. The transgenic *Tg(fli1: gfp)* line, in which endothelial vasculature displayed GFP under the control of tissue specific *Fli1* promoter (ERGB), was used to assess vascular injury and regeneration. Zebrafish embryos at 1–2 cell stage of development were collected for micro-injections. Anti-sense morpholino oligonucleotide (MO) against the ATG site of *p53* (0.5–1.0 mM, GeneTools LLC, OR) was utilized as the standard control against cytotoxic damage from MO injection (29). In addition to the control *p53* MO, erythropoietin (*epo)* mRNA (10–20 pg/nl) or *Gata1a* MO (1 mM, GeneTools LLC, OR) were respectively micro-injected to manipulate the level of hematopoiesis and subsequent viscosity-mediated WSS. *NICD* or dominant negative (DN)-*Notch1b* mRNAs (10–20 pg/nl) were used to modulate global Notch activation. Anti-sense *ephrinb2a* and *EphB4* MOs (0.5–1.0 mM, GeneTools LLC, OR) and custom-designed *ephrinb2* mRNA (10–20 pg/nl) were micro-injected to manipulate global ephrinb2 expression. Immediately after micro-injection, embryos were cultivated at 28.5°C for 3 days in fresh standard E3 medium supplemented with 0.05% methylene blue (Sigma Aldrich, MO) and 0.003% phenylthiourea (PTU, Sigma Aldrich, MO) to suppress fungal outbreak and prevent melanogenesis. At 3 days post fertilization (dpf), embryos were randomly selected for tail amputation as previously described (22). Following tail amputation, embryos were micro-injected with 6% hydroxyethyl hetastarch (Sigma Aldrich, MO) before being returned to fresh E3 medium or E3 medium dosed with pharmacological inhibitors. These inhibitors included γ-secretase inhibitor (DAPT, 100 μM), isoproterenol (100 μM), and 2,3-butanedione monoxime (BDM; 100 μM, Sigma Aldrich, MO). At 4 days post-tail amputation (dpa), dual channel confocal imaging was performed to assess vessel regeneration as previously described (13). Z-scanned images were projected in the visualization plane where voxels displayed maximum intensity. Table 1 provides the sequences of all MOs used in this study.

**Table 1:** Sequencing information of morpholino oligonucleotides (MOs).

**MOs**	**Sequence (5^′^−3^′^)**
Zebrafish *p53* MO	GCGCCATTGCTTTGCAAGAATTG
Zebrafish *Gata1a* MO	CTGCAAGTGTAGTATTGAAGATGTC
Zebrafish *ephrinb2a* MO	AATATCTCCACAAAGAGTCGCCCAT
Zebrafish *EphB4* MO (Splice)	CTGGAAAACACACACGAGAGATAGA

### 6% Hydroxyethyl Hetastarch Injection *via* Common Cardinal Vein

Double transgenic *Tg(fli1:gfp; gata1:ds-red)* embryos were immobilized with neutralized tricaine (Sigma Aldrich, MO) in 3% agarose to perform 6% hydroxyethyl hetastarch injection. The common cardinal vein (CCV) and injection site were located anatomically. The injection was recapitulated under inverted immunofluorescence microscope (Olympus, IX70) by co-injecting fluorescein isothiocyanate conjugated dextran (FITC-dextran, Sigma Aldrich, MO). Supplementary Figure 6A shows distribution of FITC-dextran post-6% hydroxyethyl hetastarch injection. Average heart rate was manually assessed at 1 h post-injection. To quantify changes in volume and plasma viscosity, sequential images of aortic flow (*ds-red*^+^) were processed to generate binary images by using ImageJ (NIH, MD). Red and blue boxes in Supplementary Figure 6C depict regions of interest to measure variations of viscosity (%, total length of *ds-red*^+^ per unit length of DA, red boxes) and flow rate (numbers of *ds-red*^+^ per unit length of DA, blue boxes) following 6% hydroxyethyl hetastarch injection. By using cultured human aortic endothelial cells (HAECs), endogenous expression of Notch-related genes under static condition was assessed following 4 and 12 h of 6% hydroxyethyl hetastarch treatment.

### Assessment of Spatiotemporal Variations in Endothelial *tp1* Activity

The transgenic *Tg(tp1: gfp)* line was crossbred with the *Tg(flk1:mCherry)* line to visualize the activity of the Rbp-J? responsive element (Epstein Barr Virus terminal protein 1, *tp1*) in the vascular endothelial network. In response to genetic and pharmacologic manipulations of WSS or global Notch activity, spatiotemporal variations in endothelial *tp1* were sequentially imaged with dual channel confocal microscopy (Leica SP8, Germany) for 4 consecutive days post-tail amputation. Acquired images were superimposed and analyzed by ImageJ.

### Whole Mount Zebrafish Immunofluorescence Staining

Following tail amputation, *Tg(flk1: mCherry)* zebrafish embryos were fixed in 10% neutral buffered formalin solution (Sigma Aldrich, MO), dehydrated in methanol (Thermofisher, MA), and permeabilized in ice cold pure acetone (Sigma Aldrich, MO). Following permeabilization, zebrafish embryos were washed and blocked with 3% bovine serum albumin (Sigma Aldrich, MO) in 0.2% phosphate buffer saline + Triton-X (PBST, Sigma Aldrich, MO). Primary antibodies anti-collagen 4 (Ab6586, Abcam), anti-ephrinb2 (Ab150411, Abcam), and anti-phosphorylated histone H3 (Ser10) (06-570, Sigma Aldrich, MO) were used to detect corresponding protein expression and cell proliferation (30). Following overnight incubation, anti-rabbit (ab150077, Abcam) or anti-mouse IgG (ab150113, Abcam) conjugated to Alexa-488 was used to amplify primary-specific fluorescence.

### Quantitative Real-Time Polymerase Chain Reaction Analyses

Total RNA was purified with Bio-Rad total RNA kit (Bio-Rad, CA) and reverse-transcribed to complementary DNA (cDNA) using an iScript cDNA synthesis kit (Bio-Rad, CA) (26). Polymerase Chain Reaction (PCR) was performed using qPCR master-mix (Applied Biological Materials Inc., Canada). The primer sequences are listed in Table 2. The expression of individual target mRNAs was normalized to human actin expression.

**Table 2:** Sequencing information of qRT-PCR primers.

**Primers**	**qRT-PCR primer sequence (5^′^−3^′^)**	
Human	Forward	CGACAGGTGCAGGTGTAGC
*Dll4*	Reverse	TACTTGTGATGAGGGCTGGG
Human	Forward	CAAAGTGTGCCTCAAGGAGTATCAGTCC
*Jag1*	Reverse	GAAAGGCAGCACGATGCGGTTG
Human	Forward	TGAGCCAGCTGAAAACACTG
*Hes1*	Reverse	GTGCGCACCTCGGTATTAAC
Human	Forward	GTTCGGCTCTAGGTTCCATGT
*Hey1*	Reverse	CGTCGGCGCTTCTCAATTATTC
Human	Forward	GCGACCTGTCCTACGCCGACCTCA
*FOXO1*	Reverse	CCTTGAAGTAGGGCACGCTCTTGACC

### Batch Processing of Fluorescence Images to Examine Endothelial-Specific Expression

To quantify co-localizations of fluorophores, multi-level thresholds based on Otsu's method were used to segment single image slices in each fluorescent channel (31). The threshold level for each channel was manually selected to extract the most accurate binary masks of each slice. Pixels that represented vascular endothelium (*flk1*^+^) and the protein of interest were flagged with a value of 1, while the remaining region was regarded as background and flagged with a value of 0. Two channels of corresponding segmented images were merged together to generate the overlapping masks. Supplementary Figure 4A depicts a schematic representation of the current method. To reduce intrinsic autofluorescence from the tissue, we pre-defined the effective area of mask from 20 to 500 pixels, that is, from 25.8 to 1,290 μm^2^. The image post-processing, segmentation, and rendering were processed by MATLAB (Mathworks, MA) and ImageJ.

### Imaging Blood Flow to Assess the Formation of Vascular Lumen During Regeneration

An inverted immunofluorescence microscope (Olympus, IX70) and digital charged-coupled device (CCD) camera (QIclick, Teledyne Qimaging, Canada) was used to sequentially image vascular injury and regeneration in the presence of blood flow. Images were superimposed by using ImageJ and Corel Imaging Software (ON, Canada).

### Measuring Blood Flow Velocity and Viscosity

To measure the blood flow velocity and viscosity in each Se vessel, we took image sequences at 20–40 frames per second and, for each sequence, manually traced the center line of each Se vessel without distinguishing between SeAs and SeVs. We analyzed vessel flow using codes custom written in MATLAB. The code detects blood cells (*ds-red*^+^) in each vessel by locating points with peak intensity. Peaks that were closer than the radius of the *ds-red*^+^ (~3 μm) were coalesced into one cell at the centroid of the coalesced peaks. The number density of *ds-red*^+^ was calculated by dividing the number of *ds-red*^+^ by the length of the vessel. To obtain the velocity, *ds-red*^+^ detection was carried out in two consecutive frames. For each *ds-red*^+^ that was detected in the first frame, the closest *ds-red*^+^ in the second frame was identified. If no *ds-red*^+^ is found within 30 μm, the velocity of the *ds-red*^+^ was not calculated. Since blood flow can only have one direction in a Se vessel, velocities in all frames were calculated after *ds-red*^+^, and the flow direction of each vessel is determined by majority rule. Lastly, *ds-red*^+^ velocities that counter the overall flow direction were removed.

### Endothelial WSS Calculation

To calculate the WSS in each Se vessel, we separated regions adjacent to detected *ds-red*^+^ from the rest of the vessel. For the parts of the Se vessel that do not contain an *ds-red*^+^ the WSS was calculated as


(1) $$\begin{array}{r} \sigma_{p}=\frac{4\mu v}{r} \end{array}$$

where μ is the viscosity, *v* is the median velocity of any detected *ds-red*^+^, and *r* is the radius of the vessel. For the part of the vessel adjacent to *ds-red*^+^, we used a model that increments the total resistance of the vessel by a constant, α_*c*_, for each *ds-red*^+^ contained in the vessel (32). The occlusive strength gives an increment on vessel resistance for each *ds-red*^+^ in the vessel. From force balance between pressure drop across the vessel and the shear force, and the shear force from the plasma part of the vessel given by the Poiseuille flow, the shear stress of the *ds-red*^+^ part of the vessel is as follows:


(2) $$\begin{array}{r} \sigma_{ds-red}=\frac{4\mu v}{r}+\frac{\pi r^{3}v\alpha_{c}}{2l_{ds-red}} \end{array}$$

where *l*_*ds*−*red*_ is the length of *ds-red*^+^. In this context, we used occlusive strengths, α_*c*_, previously measured for 4 dpf wild type fish, (32) which decreases from the mid-trunk to the tail. We also used a uniform radius of 3 μm across all Se vessels, *l*_*ds*−*red*_ = 6 μ*m*, and μ= 10^−3^ Pa·s.

### Quantification of Vascular Regeneration

Vessel segmentation and area quantification of the regenerated vascular loop were performed using Amira^TM^ 3D imaging software (Thermofisher, MA). Supplementary Figure 13 shows representative images. The entire vascular network in the posterior tail segment (purple) was automatically segmented, whereas a loop of regenerated vessel (pink) was manually assessed. The number of pixels was evaluated for statistical comparison.

### Preparation of mRNAs for *in vivo* Rescue Experiments

Rat *NICD*, zebrafish *epo*, and DN-*Notch1b* cDNA were prepared as previously described (12, 22). For transient ectopic overexpression of *ephrinb2* mRNA, zebrafish ephrinb2 cDNA was amplified from zebrafish cDNA with primers and cloned into pCS2^+^ at EcoRI/Xhol sites. Clones containing the insert were selected by PCR screening, and the clones were validated by sequencing. *In vitro* transcription was performed by using mMessage SP6 kit (Thermofisher, MA). For *in vivo* rescue experiments, transcribed mRNAs were purified by using a total RNA isolation kit (Bio-Rad, CA). The sequences of cloning primers are listed in Table 3.

**Table 3:** Cloning primers.

**Primers**	**qRT-PCR primer sequence (5^′^−3^′^)**	
Rat	Forward	GCAGGATCCACCATGGGTTGTGGGGTGCTGCTGTCCCGCAAG
	Reverse	CTTGAATTCTTACTTAAATGCCTCTGGAATGTGGGTG
Zebrafish	Forward	GATCCCATCGATTCGAATTCACCATGCATCTTTTCTTCGTGAAACTAATTGTTG
*DN-Notch1b*	Reverse	CTATAGTTCTAGAGGCTCGAGCTAAGCGTAATCTGGAACATCGTATGGGTATTCTCCGACCGGCTCTCTCCTC
Zebrafish	Forward	ATTCGAATTCCACCATGGGCGACTCTTTGTGGAGATATTACTTTG
*Ephrinb2*	Reverse	AGAGGCTCGAGTCACACCTTGTAATAGATGTTTGCTGGGC

### Small Interference RNA Transfection to Cultured Endothelial Cells

Primary HAEC (Cell Applications, CA) were grown on bovine gelatin (Sigma Aldrich, MO)-coated-plates (Midsci, MO) at 37°C and 5% CO_2_ and propagated for experiments between passages 4 and 10. Endothelial cell (EC) growth medium (Cell Applications, CA) was supplemented with 5% fetal bovine serum (FBS, Life technologies, NY) and 1% penicillin-streptomycin (Life Technologies, NY) for optimal EC cultivation. At ~50% confluency, FlexiTube^TM^ siRNAs targeting scrambled negative control (Scr), Notch1, or ephrinb2/ EphB4 (Qiagen, Germany) were transfected following manufacturer's instruction. Lipofectamine^TM^ RNAiMAX (Thermofisher, MA) diluted with dulbecco's modified eagle medium (DMEM)/10% FBS and Opti-MEM media with reduced serum (Thermofisher, MA) was used for siRNA transfection. Efficacy of transfections was verified by immunoblotting.

### EC Migration and Matrigel Tube Formation Assay

Human aortic endothelial cell (HAEC) migration assay was performed as previously described (13). Areas between inner borders of HAEC at 4, 6, 12, 24 h post scratch were evaluated using ImageJ. A tube formation assay was performed by seeding HAEC in a 96-well plate coated with Matrigel with reduced growth factors (BD Biosciences, CA) in the presence of human vascular endothelial growth factors (VEGF; 10–20 ng/ml, Sigma Aldrich, MO). Following 4 h of incubation at 37°C, tube formation was evaluated under an Olympus IX 70 phase-contrast microscope. The number of branching points and tube length were manually quantified using ImageJ.

### Pulsatile Shear Stress Exposure

Confluent monolayers of HAEC grown on a 6-well plate were exposed to unidirectional pulsatile shear stress (PSS; 6, 12, and 24 h) using a modified flow device (14). A neutralized MCDB-131 medium (Sigma Aldrich, MO) containing 7.5% sodium bicarbonate solution, 10% FBS, and 4% dextran from *Leuconostoc spp* (Sigma Aldrich, MO) was used for PSS exposure. Following exposure to PSS, the center of each monolayer was removed by using a cell scraper to collect only flow-aligned cells from the periphery of the well. To visualize changes in endogenous protein expressions and proximity ligations, we utilized our in-house dynamic flow system (∂τ/∂t = 29.3 dyne·cm^−2^·s^−1^, with time-averaged shear stress = 50 dyne·cm^−1^ at 1 Hz) (33).

### Immunoprecipitation, Proximity Ligation Assays, and Immunoblot Analysis

Following PSS exposure, flow-aligned cells were lysed with mammalian protein extraction reagent (M-PER; Thermofisher, MA) supplemented with 1% protease and phosphatase inhibitor cocktail (Thermofisher, MA) at 4°C. Detergent compatible protein assay (Bio-rad, CA) and lithium dodecyl sulfate polyacrylamide gel electrophoresis (Invitrogen, CA) were performed as previously described (34). Primary antibodies anti-Notch1 (MA5-32080, Thermofisher, MA), anti-ephrinb2 (Ab150411, Abcam), and anti-EphB4 (H-200, Santa Cruz Biotechnology Inc., TX) were used. Equal loading was verified by using anti-β-tubulin (AA2, Santa Cruz Biotechnology Inc., TX). Anti-rabbit (7074S, Cell Signaling Technology) or anti-mouse IgG (7076S, Cell Signaling Technology) conjugated to horseradish peroxidase was used for the secondary incubation. Immunoprecipitation against ephrinb2/EphB4 was performed using a Pierce^TM^ Crosslink magnetic IP/Co-IP kit (Thermofisher, MA). Disuccinimidiyl suberate was used to crosslink anti-ephrinb2 which was neutralized for immunoblot analyses. Densitometry was performed as previously described (26). Proximity ligations between ephrinb2 and EphB4 in flow-aligned cells were conducted using Duolink^?^ *In situ* Red Starter Kit Mouse/Rabbit (Sigma Aldrich, MO). Numbers of individual ligations in raw and post-processed images were compared to test the validity of the quantification. To evaluate polarization kinetics and distributions of the ligations, platelet endothelial adhesion molecule 1 (H-3, Santa Cruz Biotechnology Inc., TX) and 4′,6-diamidino-2-phenylindole (SC-3598, Santa Cruz Biotechnology Inc., TX) were fluorescently labeled.

### Statistics

Data were expressed as mean ± standard deviation and compared among separate experiments. Unpaired two-tail *t* test and 2-proportion *z-*test were used for statistical comparisons between 2 experimental conditions. *P* values < 0.05 were considered significant. Comparisons of multiple values were made by one-way analysis of variance (ANOVA), and statistical significance for pairwise comparison was determined by using the Tukey test.

## Results

### Tail Amputation Increases Peak WSS in the SeA Closest to the Amputation Site to Promote Notch-Mediated DLAV-PCV Loop Formation

**Figure 1:**
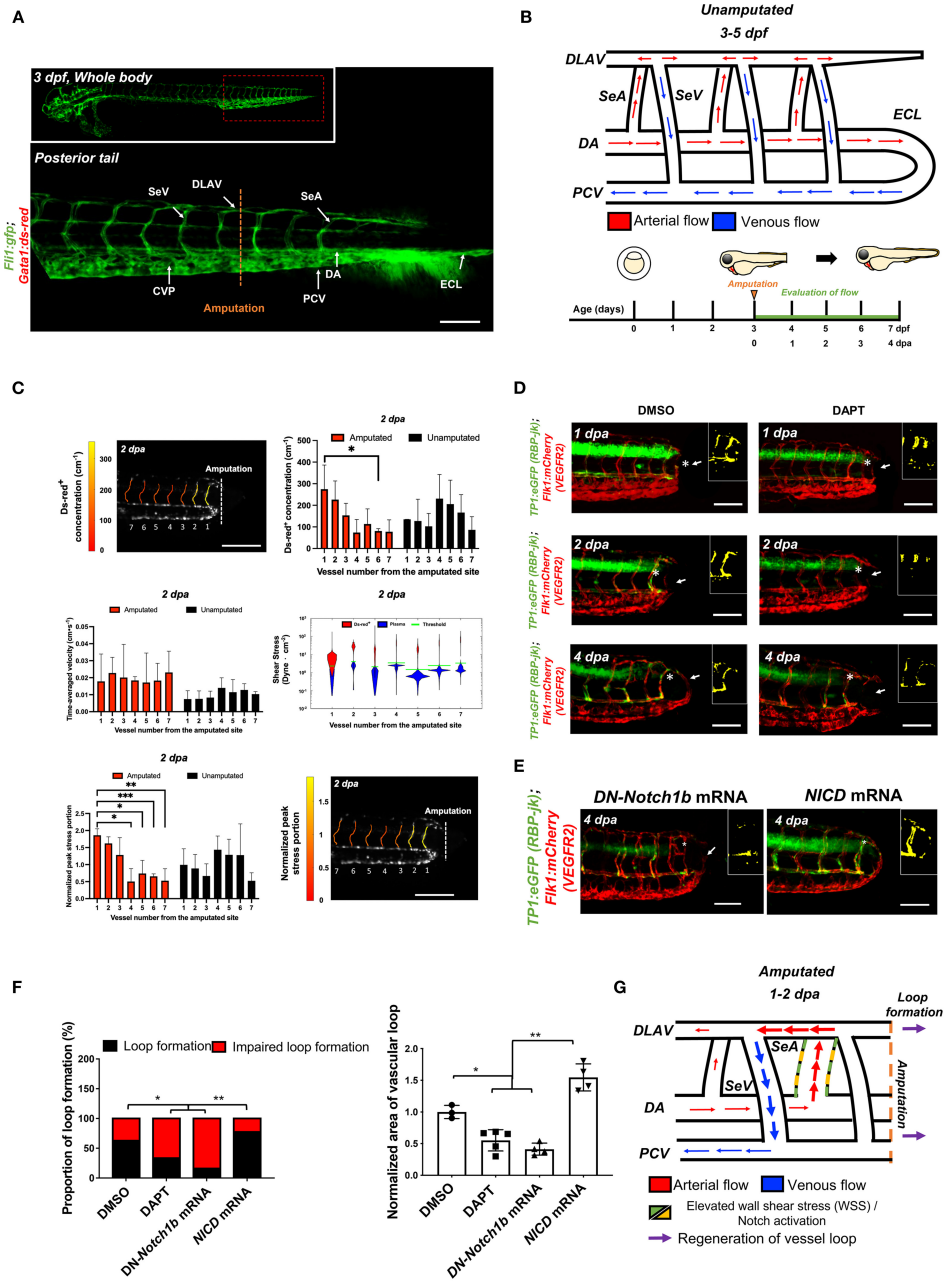
Tail amputation increases peak wall shear stress (WSS) in the segmental artery (SeA) closest to the amputation site to promote Notch-mediated dorsal longitudinal anastomotic vessel-posterior cardinal vein (DLAV-PCV) loop formation. **(A)** Anatomy of tail vasculature in *Tg(fli1:gfp; gata1:ds-red)* embryos; Scale bar: 100 μm. **(B)** Wiring diagram illustrate embryonic circulation in zebrafish tail. In the caudal vascular network, dorsal aorta (DA) forms an embryonic circulatory loop (ECL) with PCV (white arrow), and venous flow in the DLAV and venous segmental vessel (SeV) drain into PCV (white arrowheads). Experimental design: At 3 days post fertilization (dpf), embryos were randomly chosen for tail amputation (~100 μm of posterior tail segment). Hemodynamic WSS was evaluated for 4 consecutive days. **(C)** At 2 dpa, *ds-red*^+^ concentration (cm^−1^, number of *ds-red*^+^ per unit length of SeA) in the amputated site increased significantly compared to unamputated embryos (Mean ± standard deviation across the vessels in 3 unamputated embryos, averaged over 3.6 mins). Time-averaged velocity (cm·s^−1^) remained unchanged. The distribution of WSS exerted by *ds-red*^+^ (red) is shifted upward compared to that exerted by plasma (blue), giving intermittent rises of WSS as each *ds-red*^+^ passes. Although this separation is present in every SeA, the portion of ECs (red area) experiencing WSS from *ds-red*^+^ is higher in the proximal SeAs than in the distal SeAs, leading to a higher peak stress portion. The areas scale with but are not equal to the portion of endothelial cells (ECs) that experience stress from *ds-red*^+^ and plasma. Green line shows the 0.975 percentile of WSS from plasma, which is used as an activation threshold. For the calculation of WSS, see Methods. The increase in viscosity resulted WSS to exceed activation threshold in the site of amputation. The threshold is set to 0.975 percentile of plasma shear stress across all intersegmental vessels, and ranges from 1.1 to 5.4 dyne·cm^−2^. **(D)** In the transgenic *Tg(tp1:gfp; flk1: mcherry)* line, vehicle Dimethyl sulfoxide (DMSO)-treated zebrafish showed prominent endothelial *tp1* activity in the proximal SeA (*asterisk, overlapped yellow) and formed a new vascular loop between the DLAV and PCV at 4 dpa (white arrow). Pharmacological γ-secretase inhibitor (DAPT) treatment (100 μM) attenuated endothelial *tp1* activity in the amputated site and neighboring SeA and impaired regeneration of the loop by 4 dpa (white arrow). Scale bar: 20 μm. **(E)** Transient suppression of Notch activity via DN-*Notch1b* mRNA, but not Notch Intracellular Cytoplasmic Domain (*NICD)* mRNA, impaired vascular loop formation. Scale bar: 20 μm. **(F)** Quantification of the proportion of embryos exhibiting loop formation and normalized area of vascular loop (^*^p< *0.05* vs. DMSO, ^**^p< *0.005* vs. *NICD* mRNA, *n* = 17 for DMSO, *n* = 20 for DAPT, DN-*Notch1b* and *NICD* mRNAs to assess proportion of loop formation, *n* = 3 for DMSO, *n* = 5 for DAPT, DN-*Notch1b* and *NICD* mRNAs to assess area of vascular loop). **(G)** Wiring diagrams illustrate amputation-mediated changes in blood flow and hemodynamic WSS. SeA, Arterial segmental vessel; SeV, Venous segmental vessel; DLAV, Dorsal longitudinal anastomotic vessel; PCV, Posterior cardinal vein; DA, Dorsal aorta; CVP, Caudal vein capillary plexus; ECL, Embryonic circulatory loop. ^***^p < 0.0005.

To assess the effect of amputation on microvascular flow, we used the double transgenic *Tg(fli1:gfp; gata1:ds-red)* zebrafish line to simultaneously visualize the vascular network (*gfp*^+^) and blood cells (*ds-red*^+^). Prior to tail amputation, arterial blood passes through the DA either to a set of parallel SeAs that drain into the DLAV and then into the PCV via SeVs, (32) or directly to the PCV via an anastomosis at the caudal end of the trunk (Figures 1A,B). (35) Following tail amputation at 3 dpf, hemodynamic changes in the distal SeAs and SeVs were evaluated for 4 consecutive days (Figure 1B). We used custom-coded tracking software (see Methods) to track the individual *ds-red*^+^ and measure the time-average velocity of *ds-red*^+^ and WSS in the amputated region (*n* = 3) (32). Tail amputation severed circulation through the ECL, causing *ds-red*^+^ to be routed through nearby SeAs (Figure 1C, Supplementary Figure 1). In particular, *ds-red*^+^ concentration increased by 3.6-fold in the SeA closest to the amputation site, but only 2.2-fold in the third closest SeA, whereas velocity remained unchanged throughout the trunk. Average WSS in the segmental vessels decreases from head to tail as defined by the pressure drop across the vessel and its cross-section area. Cross-section areas vary little between vessels, and pressures decrease along the DA. However, *ds-red*^+^ and SeA radii closely conform, thus, WSS near *ds-red*^+^ are large, and our modeling reveals that the passage of *ds-red*^+^ along a SeA is accompanied by a traveling pulse of high WSS. We accounted for the heterogeneous WSS by the *peak shear stress portion*, the fraction of time during which the WSS exceeds a certain threshold, and found that the increase in *ds-red*^+^ concentration proximal to the amputation site produced an increase in *peak shear stress portion*, but only within this SeA (Figure 1C). Increased flow in the proximal SeA triggers two forms of network plasticity: remodeling of the SeA and formation of new vessels from the DLAV that reconnected DA and PCV. We observed that the SeA radius increased over 1 dpa with no further change afterward (Supplementary Figure 2). This finding is consistent with the WSS set point model, predicting that when the WSS in a vessel is larger than its set point, its radius will increase until the stress set point is retained by 4 dpa.

Next, we set out to demonstrate that these topological changes following injury are directly linked to the WSS changes within the SeA to drive Notch signaling when vessels are newly formed. Following tail amputation, transgenic *Tg(tp1:gfp; flk1: mcherry)* embryos displayed prominent endothelial *tp1* activity (color-coded to accentuate colocalized Notch activity in the endothelial vasculature) in the SeA close to the injury site, accompanying the formation of the new vascular loop (Figure 1D). Inhibiting Notch signaling by treating with DAPT attenuated endothelial *tp1* activity in the SeAs and impaired the formation of the DLAV-PCV loop at 4 dpa (Figures 1D,F). Local endothelial *tp1* activity and loop formation were also suppressed by micro-injecting DN-*Notch1b* mRNA. Conversely, transient ectopic overexpression of *NICD* mRNA upregulated *tp1* activity in the injured SeA to promote DLAV-PCV loop formation. (^*^p< *0.05* vs. Dimethyl sulfoxide (DMSO), ^**^p< *0.05* vs. DN-*Notch1b* mRNA, *n* = 17 for DMSO and *n* = 20 for DAPT to assess proportion of loop formation, *n* = 3 for DMSO and *n* = 5 per other groups to assess area of vascular loop) (Figures 1E,F). A new lumenized vascular loop formed with increased collagen 4 (*ColIV*) basal lamina deposition both in the proximal SeA and in regenerated vessels. This expression was attenuated by DAPT treatment (Supplementary Figure 3). Amputation-mediated Notch signaling and vascular regeneration is summarized in Figure 1G.

In addition, we performed migration assays with endothelial cells *in vitro* to recapitulate the pathological microenvironment cues. Pharmacological inhibition of Notch cleavage using DAPT treatment and silencing Notch1 expression with siRNA (*siNotch1*) reduced endothelial migration and Matrigel tube formation (Supplementary Figures 4A–F). Taken together, our *in vivo* and *in vitro* findings support the role of Notch signaling in EC migration for vascular loop formation after injury.

### Changes in WSS Modulate DLAV-PCV Loop Formation in a Notch-Dependent Manner

**Figure 2:**
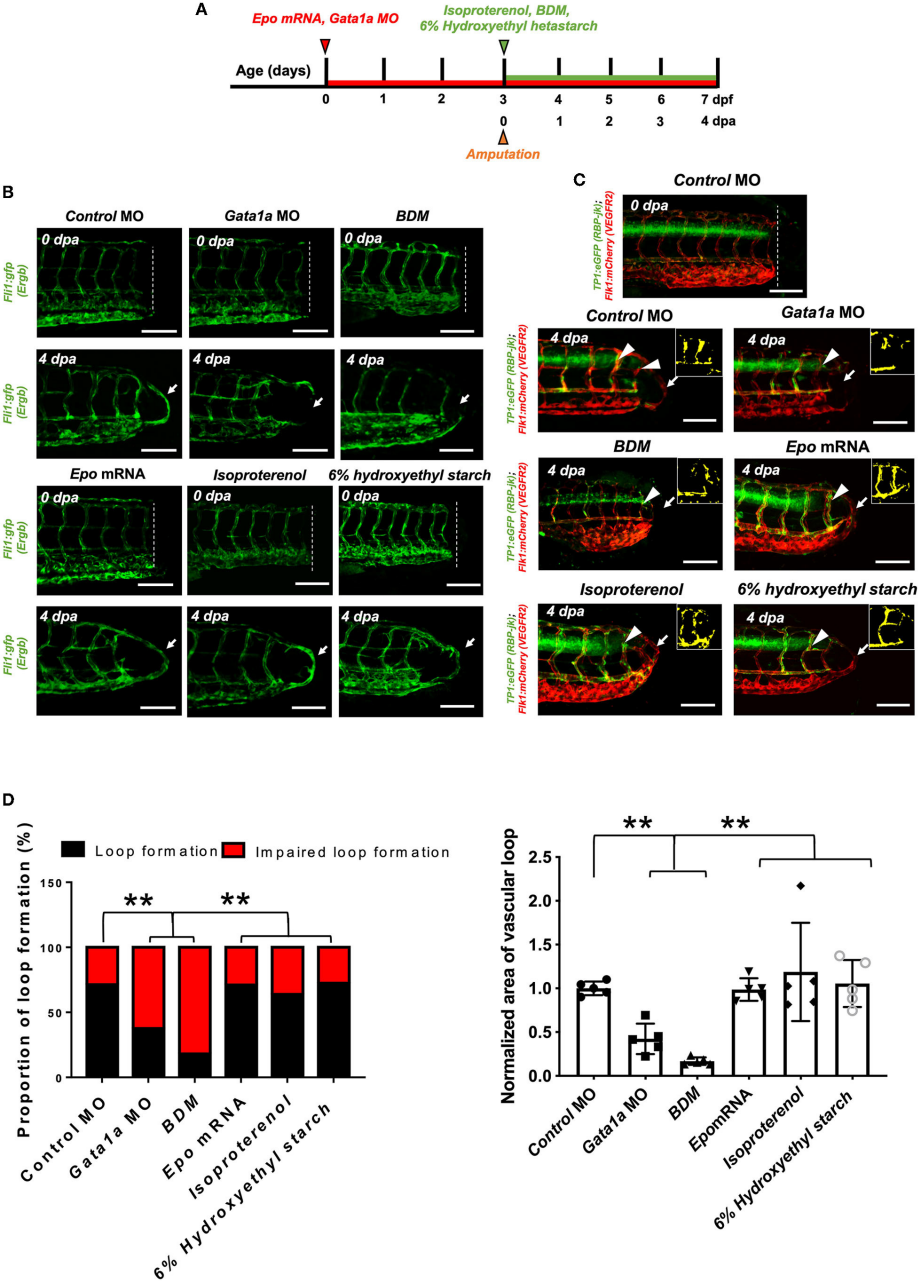
Changes in WSS modulate DLAV-PCV loop formation in a Notch-dependent manner. **(A)** Experimental design to genetically and pharmacologically manipulate hemodynamic WSS in zebrafish embryos. **(B)** In response to tail amputation, *Gata1a* morpholino oligonucleotides (MO) injection (1 mM) or 2,3-butanedione monoxime (BDM, 100 μM) treatment impaired vascular loop formation at 4 dpa (white arrows). Erythropoietin (*epo*) mRNA injection (10–20 pg/nl), isoproterenol treatment (100 μM) and 6% hydroxyethyl hetastarch promoted regeneration and vascular loop formation. Scale bar: 20 μm. **(C)** *Gata1a* MO and BDM treatment reduced endothelial *tp1* activity and impaired regeneration, whereas *epo* mRNA injection or isoproterenol treatment up-regulated endothelial *tp1* activity in the amputated site (white arrowheads) and promoted vascular loop formation at 4 dpa (white arrows). Scale bar: 20 μm. **(D,E)** Quantification of the proportion of embryos exhibiting loop formation and normalized area of vascular loop (^*^p< *0.05*, ^**^p< *0.005* vs. *control* MO, *n* = 20 for each group to assess proportion, *n* = 5 for each group to assess area of vascular loop). Scale bar: 20 μm.

To test whether formation of a new vascular loop occurs in response to WSS triggers, we manipulated WSS by transiently modulating zebrafish blood viscosity (since *peak stresses* reflect the flux of *ds-red*^+^ through a vessel) or myocardial contractility (Figure 2A, Supplementary Figure 5). Reduction in hematocrit and, therefore, viscosity via *Gata1a* MO injection or inhibition of myocardial contractility with BDM (36) impaired the DLAV-PCV loop formation at 4 dpa. Conversely, augmenting hematocrit via *epo* mRNA injection or increasing contractility via isoproterenol treatment promoted loop formation at 4 dpa (^**^p< *0.005* vs. *control* MO, *n* = 20 per group to assess proportion, *n* = 5 for each group to assess area of vascular loop) (Figures 2B,D,E). Consistent with these findings, increasing plasma viscosity by introducing 6% hydroxyethyl hetastarch via the common cardinal vein (CCV) (~5.5 % volume expansion) promoted DLAV-PCV loop formation without influencing the average heart rate (*n* = 5, averaged 3.6 mins), flow rate of *ds-red*^+^ (*n* = 4, averaged 3 cardiac cycles), and endogenous expression of Notch-related genes (^**^p< *0.005*, ^***^p< *0.0005* vs. H_2_O, normalized to human actin, *n* = 3) (Figure 2B, Supplementary Figure 6) (37). Next, we monitored the effects of modulating hemodynamics on *tp1* activity. At 2 dpa, both *Gata1a* MO injection and BDM treatment reduced endothelial *tp1* activity compared to the *control* MO-injected embryos. Transient *epo* mRNA overexpression or isoproterenol treatment and injection of 6% hydroxyethyl hetastarch upregulated endothelial *tp1* activity at 2 dpa. (Figures 2C,D) Modulation of both WSS and global Notch activity (DAPT treatment and *NICD* and DN-*Notch1b* mRNA injections) corroborated WSS-mediated Notch signaling to promote DLAV-PCV loop formation (^*^p< *0.05* vs. *NICD* mRNA + *Gata1a* MO, *n* = 4 per group) (Supplementary Figure 7). As a corollary, PSS (∂τ/∂t = 23 dynes·cm^−2^ at 1 Hz) upregulated, while DAPT treatment mitigated Notch signaling-related gene expression in HAECs (Supplementary Figure 4G).

**Figure 3:**
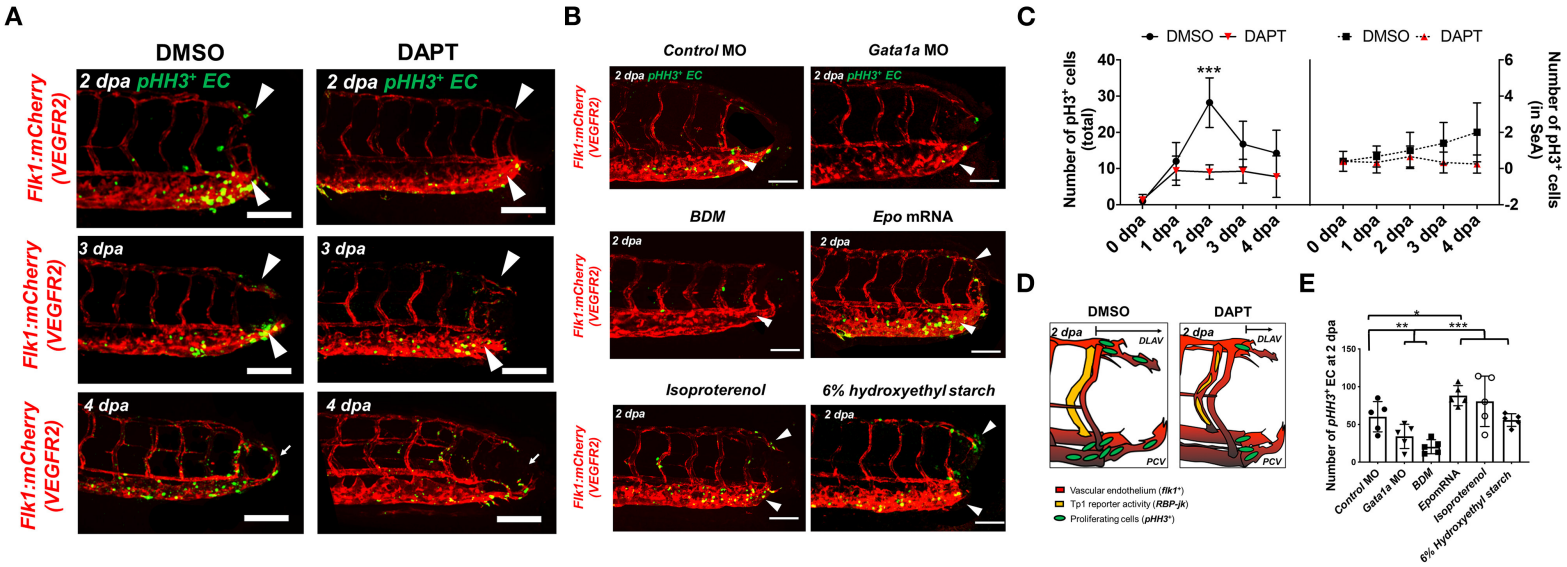
Amputation-mediated Notch signaling regulates EC proliferation in the DLAV and CVP during vascular loop formation. **(A)** DAPT treatment to inhibit Notch activity significantly reduced the number of EC proliferation (*pHH3*^+^EC, green) compared to DMSO-treated controls at 2 dpa (white arrowheads). Scale bar: 20 μm. **(B)** *Gata1a* MO injection or BDM treatment reduced, whereas *epo* mRNA and isoproterenol treatment increased *pHH3*^+^ EC at 2 dpa. (^*^p< *0.05*, ^**^p< *0.005*, ^***^p< *0.0005* vs. *control* MO, *n* = 5 for each group). Scale bar: 20 μm. **(C)** Total numbers of endothelial *pHH3*^+^ EC in posterior tail segment and the caudal SeA were assessed to quantify Notch-dependent proliferation. 2 days of DAPT treatment resulted ~48% reduction in total number of *pHH3*^+^ EC, but not in the caudal SeA (^**^p< *0.005* vs. DMSO, *n* = 3 for DMSO, *n* = 5 per other groups). **(D)** Schematic representation of Notch-mediated *pHH3*^+^ EC during vascular loop formation. **(E)** Total *pHH3*^+^ EC in posterior tail were assessed to quantify WSS-dependent EC proliferation. *Gata1a* MO injection or BDM treatment reduced ~42 and ~65%, whereas *epo* mRNA and isoproterenol treatment increased *pHH3*^+^ EC by ~52 and ~39% respectively at 2 dpa. (^*^p< *0.05*, ^**^p< *0.005*, ^***^p< *0.0005* vs. *control* MO, *n* = 5 for each group). Scale bar: 20 μm.

Both WSS and Notch signaling are critical regulators of EC proliferation (14, 38). Next, we assessed whether amputation-mediated Notch activity regulates EC proliferation for vascular loop formation. Colocalization of vascular endothelium (*flk1*^+^) and the cell mitosis marker phospho-histone 3 were assessed to evaluate EC proliferation (*pHH3*^+^ EC). While DMSO-treated embryos showed a significant increase in the number of *pHH3*^+^ EC in the caudal vein capillary plexus (CVP), DLAV, and regenerated vessels, DAPT treatment for 2 days resulted in ~48% reduction in total number *pHH3*^+^ EC, but not in the SeA adjacent to the amputated site (^***^p< *0.005* vs. DMSO, *n* = 5 per group) (Figures 3A,C). This suggested that amputation-mediated Notch activity in the SeA laterally induced EC proliferation in the DLAV and CVP (Figure 3D) (39). The number of total *pHH3*^+^ EC in the amputated site was also dependent on the level of WSS (Figures 3B,E). Reduction of WSS *via Gata1a* MO injection and BDM treatment diminished the number of total *pHH3*^+^ EC by ~42 and ~65%, respectively. Consistent with this observation, *epo* mRNA overexpression or isoproterenol treatment upregulated *pHH3*^+^ EC by ~52 and ~39%, respectively, at 2 dpa. Intriguingly, injection of 6% hydroxyethyl hetastarch increased endothelial *tp1* activity, but did not increase the number of *pHH3*^+^ EC at 2 dpa (^*^p< *0.05*, ^**^p< *0.005*, ***p< *0.0005* vs. *control* MO, *n* = 5 per group). Taken together, our findings support the idea that elevated hemodynamic WSS underlies endothelial Notch-mediated vascular loop formation.

### Partitioned Blood Flow Promotes Notch Signaling Pathway for Arterial Network Formation

**Figure 4:**
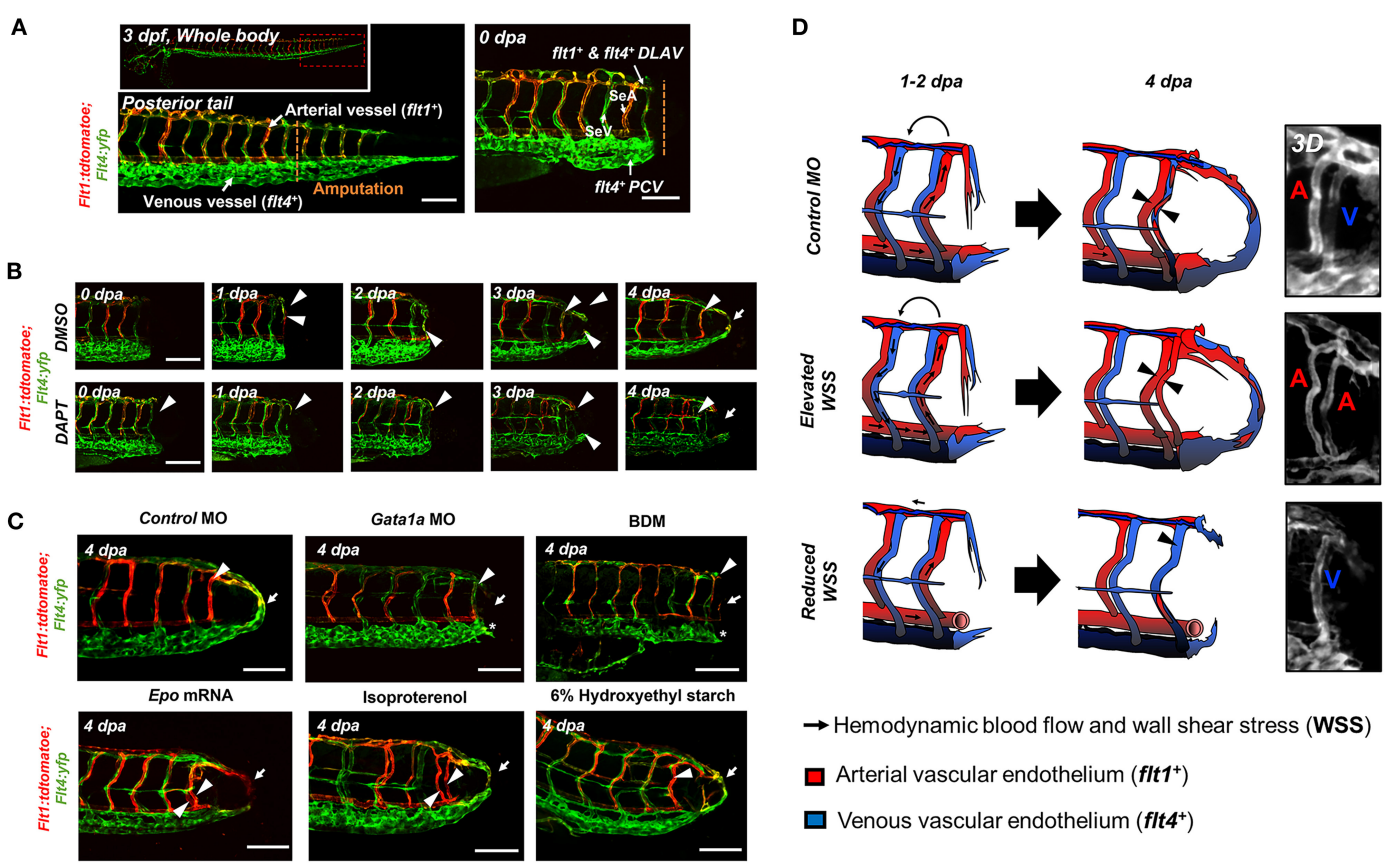
WSS promotes Notch-mediated arterial network. **(A)** A representative image of tail vasculature in the transgenic *Tg(flt1:tdtomatoe; flt4: yfp)* zebrafish line at 3 dpf. *flt1*^+^: arterial vascular endothelium, *flt4*^+^: venous vascular endothelium. *flt1*^+^ &* flt4*^+^ DLAV: *flt1*^+^ or *flt4*^+^ regenerated from the DLAV. *flt4*^+^ PCV: *flt4*^+^ regenerated from the PCV. Scale bar: 100 μm. **(B)** In response to DMSO treatment, *flt1*^+^ arteries preferentially formed an initial dorsal to ventral connection between the DLAV (*flt1*^+^ DLAV) and the DA in the amputated site (white arrowhead). Between 1–2 dpa, *flt4*^+^ veins from the DLAV (*flt4*^+^ DLAV) regenerated toward the DA exhibiting collateral arterial phenotype (overlapped yellow, white arrowheads). While regeneration of *flt4*^+^ veins occurred in the PCV (*flt4*^+^ PCV) and DLAV (*flt4*^+^ DLAV) for loop formation between 2–3 dpa, the distal segmental network exhibited enhanced *flt1* expression at 3 dpa. *flt1*^+^ arteries extended dorsally from SeA at 4 dpa and formed a vascular loop with *flt4*^+^ PCV (white arrow). Conversely, DAPT treatment inhibited the initial connections of both *flt1*^+^ arteries and *flt*4^+^ veins at 2 dpa and partially inhibited both *flt*4^+^ DLAV and CVP at 3 dpa (white arrowheads). At 4 dpa, DAPT treatment inhibited *flt1*^+^network (white arrowheads) and attenuated loop formation at 4 dpa (white arrows, *n* = 5 per group). Scale bar: 20 μm. **(C)** Following tail amputation, *Gata1a* MO injection or BDM treatment diminished *flt1*^+^ in the amputated site (white arrowheads) and partially attenuated *flt4*^+^ DLAV and PCV (*asterisk, *n* = 20 per group). Increase in WSS (*epo* mRNA, isoproterenol, 6% hydroxyethyl hetastarch) enhanced *flt1*^+^ network (white arrowheads) during loop formation (white arrows) as compared to MO-injected controls. (*n* = 20 per group). Scale bar: 20 μm. **(D)** Schematic representations and 3-dimensional (3D) overview of WSS-mediated arterial- and venous- regeneration. Black arrowheads depict regenerated *flt1*^+^ (Red) and *flt*4^+^ network (Blue) in response to changes in WSS.

To elucidate whether WSS-mediated Notch pathway influences the arterial and/or venous network formation during DLAV-PCV loop formation, we utilized the transgenic *Tg (flt1: tdtomatoe; flt4: yfp)* zebrafish line, allowing for simultaneous visualization of arterial (*flt1*^+^) and venous (*flt4*^+^) networks (Figure 4A). Time-lapse imaging in the presence or absence of DAPT treatment revealed that WSS-mediated Notch signaling regulates *flt1*^+^ network during loop formation. DMSO-treated controls developed a secondary loop of *flt1*^+^ DLAV that dorsally extended from the distal SeA in conjunction with blood flow to anastomose with *flt4*^+^ PCV (Supplementary Video 2). Treatment with DAPT inhibited the initiation of *flt1*^+^ DLAV to connect with DA (0–1 dpa) and the collateral arterialization of *flt4*^+^ DLAV (2–3 dpa) (40), resulting in an impaired *flt1*^+^ network in the amputated site at 4 dpa (Figure 4B, Supplementary Figure 8). Reducing viscosity via *Gata1a* MO injection diminished the distal *flt1*^+^ in both SeA and DLAV, and partially impaired *flt4*^+^ DLAV from forming a loop with the PCV (Figures 4C,D). BDM treatment abrogated both *flt1*^+^ and *flt4*^+^ for vascular loop formation. In contrast, both increased viscosity via *epo* mRNA or 6% hydroxyethyl hetastarch injection or increasing myocardial contractility via isoproterenol treatment promoted *flt1*^+^network formation in the amputated site as compared to MO-injected controls (Figures 4C,D). Gain- and loss-of-function analyses of global Notch activity further corroborated that WSS-activated Notch signaling coordinates *flt1*^+^*/ flt4*^+^ loop formation (*n* = 20 per group) (Supplementary Figure 9). As a corollary, genetic and pharmacologic elevation of hemodynamic WSS failed to restore loop formation in the presence of DAPT treatment or DN-*Notch1b* mRNA injection. However, *NICD* mRNA injection restored *Gata1a* MO-impaired *flt1*^+^/ *flt4*^+^ loop formation at 4 dpa (*n* = 20 per group) (Supplementary Figure 9). Taken together, our results indicate that WSS-responsive endothelial Notch signaling activates growth of the *flt1*^+^network and arterializes the *flt4*^+^ DLAV to form a new vascular loop.

### WSS-Responsive Notch-Ephrinb2 Pathway Regulates Arterial Network Formation

**Figure 5:**
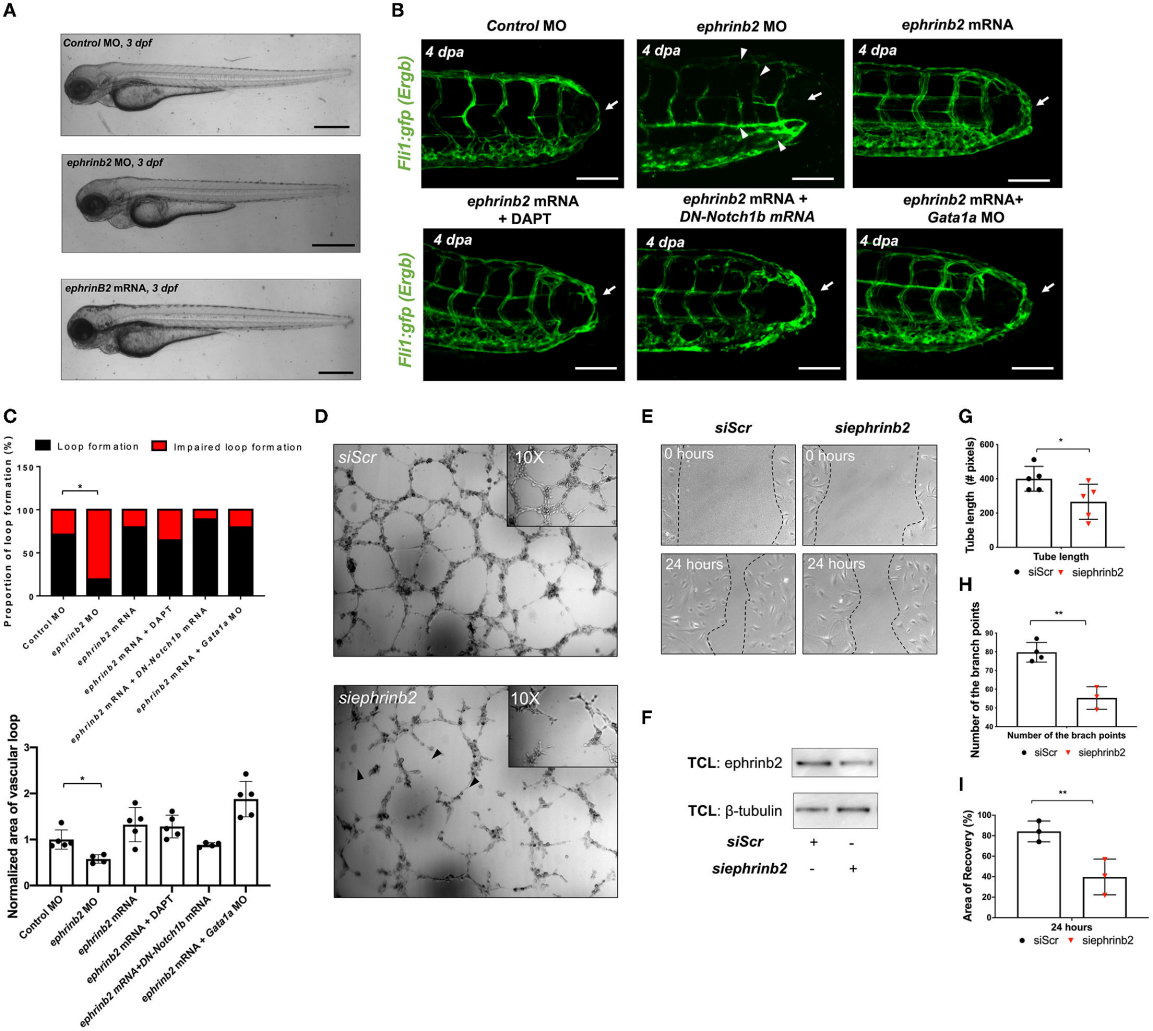
Ephrinb2 regulates vascular loop formation. **(A)** Gross morphology following global ephrinb2 modulation. Both *ephrinb2* MO and mRNA injections did not affect gross zebrafish morphology (white arrowheads). Scale bar: 100 μm. **(B)** Compared to the MO-injected controls, ephrinb2 MO (0.5–1 mM)-injected embryos had impaired loop formation (white arrow) altered the DA and SV morphology, immature CVP and DLAV at 4 dpa (white arrowheads). Transient overexpression of *ephrinb2* mRNA restored DAPT, DN-*Notch1b* mRNA (10–20 pg/nl) and *Gata1a* MO-impaired loop formation (white arrows). Scale bar: 20 μm. **(C)** Quantification of the proportion of embryos exhibiting loop formation and normalized area of vascular loop (^*^p< *0.05* vs. *control* MO, *n* = 20 per group). **(D,E)** Representative images of Matrigel and human aortic endothelial cell (HAEC) migration assays following *siScr* or *siephrinb2* transfection. **(F)** The density quantification of ephrinb2 expression following *siephrinb2* transfection. TCL: total cell lysates **(G,H)** *siephrinb2* transfection reduced both tube length and the number of branch points as compared to *siScr*-transfected HAEC (^*^p< *0.05*, ^**^p< *0.005* vs. *siScr, n* = 5 for tube length, *n* = 4 for branch point). **(I)** *siephrinb2* transfection reduced the attenuated area of recovery (A.O.R) by ~45% at 24 h post scratch (^**^p< *0.005* vs. *siScr, n* = 3 per group).

**Figure 6:**
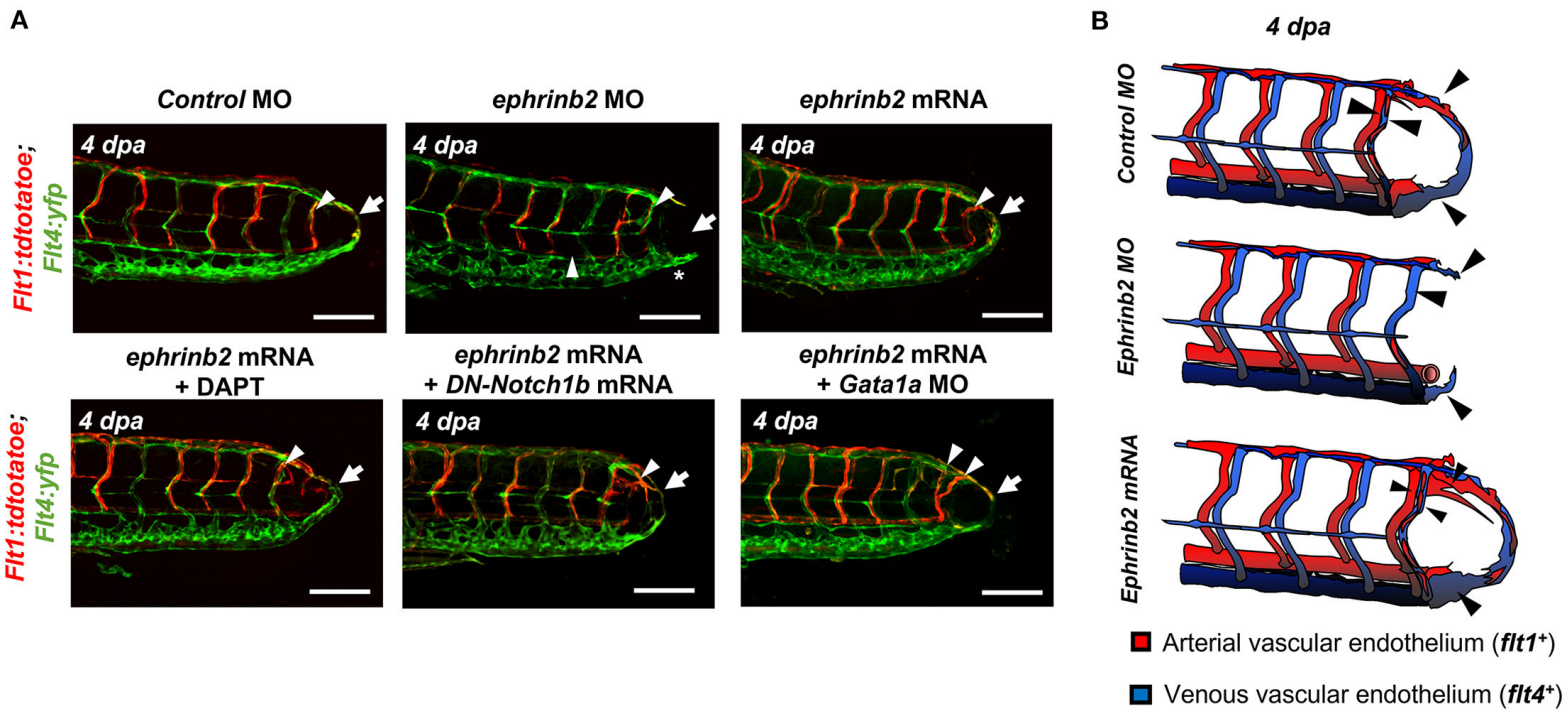
WSS-responsive Notch-ephrinb2 pathway regulates arterial network formation. **(A)** Injection of *ephrinb2* MO (0.5-1mM) diminished *flt1*^+^ in the amputated site (white arrowheads) to attenuate loop formation at 4 dpa (white arrow). Transient overexpression of *ephrinb2* mRNA promoted *flt1*^+^ network formation in the amputated site (white arrowheads) and restored DAPT and DN-*Notch1b* mRNA-impaired *flt1*^+^ regeneration (white arrow) (*n* = 20 per group). Scale bar: 20 μm. **(B)** Schematic representations of ephrinb2-dependent *flt1*^+^ and *flt14*^+^ regeneration. Black arrowheads depict regenerated *flt1*^+^ (Red) and *flt*4^+^ (Blue) in response to differential ephrinb2 expressions.

To investigate the downstream signaling pathways underlying DLAV-PCV loop formation, we performed 1) immunostaining for endogenous ephrinb2 and 2) batch processing to assess spatiotemporal variations in endothelial ephrinb2 *in situ* following tail amputation (Supplementary Figure 10A). Endothelial ephrinb2 staining increased at 1 dpa in the distal DA and DLAV where WSS-responsive arterial network formation occurs. Following the initial anastomosis between the DLAV and DA at 2 dpa, the distal SeA and regenerated vessel region expressed prominent ephrinb2 staining to form a new vascular loop at 4 dpa. *Gata1a* MO injection and BDM treatment attenuated endothelial ephrinb2 staining from 2 to 4 dpa, whereas *epo* mRNA-, isoproterenol-, and 6% hydroxyethyl hetastarch-augmented WSS accentuated staining in the distal SeA, DLAV, and CVP from 2 to 4 dpa. DAPT treatment, as the positive control, reduced ephrinb2 staining to affirm that the Notch-dependent ephrinb2 pathway is involved in forming the DLAV-PCV loop (^*^p< *0.05* vs. *control* MO *n* = 5 per group) (Supplementary Figure 10B). Consistent with these observations, in our *in vitro* experiments with cultured ECs exposed to shear stress conditions, PSS upregulated Ephrin-B2 protein in a time- and Notch-dependent manner in HAEC (^*^p< *0.05*, ^**^p< *0.005*, ^***^p< *0.0005* vs. static, *n* = 3 per time point) (Supplementary Figure 11A). As an internal positive control, Kruppel Like Factor 2 (KLF2) mRNA expression was also upregulated (^**^p< *0.005* vs. static, *n* = 3 per time point) (Supplementary Figure 11B) (14). In addition, we genetically manipulated global ephrinb2 expression via MO or mRNA injection to assess vascular loop formation in the transgenic *Tg(fli1: gfp)* zebrafish embryos. Neither knockdown nor overexpression of ephrinb2 expression resulted in gross abnormalities in zebrafish morphology (Figure 5A). Injection of *ephrinb2* MO impaired loop formation, leading to a collapsed DA morphology and maturity of the DLAV and CVP at 4 dpa. Conversely, transient ectopic overexpression of *ephrinb2* mRNA increased endoluminal sizes of regenerated vessels and restored DAPT-, DN-*Notch1b* mRNA-, and *Gata1a* MO-impaired loop formation (^*^p< *0.05* vs. *control* MO, *n* = 20 per group for proportion, *n* = 5 per group for area) (Figures 5B,C). *In vitro* Matrigel and migration assays following *siephrinb2* transfection further supported these observations (Figure 5F). While *siephrinb2* transfection resulted in ~30 and ~32% reduction in the tube length and the number of the branch points, respectively (Figures 5D,G,I), area of recovery was reduced by ~49% at 24 h post scratch (^*^p< *0.05*, ^**^p< *0.005* vs. *siScr* transfection, *n* = 5 for tube length, *n* = 4 for branch point, *n* = 3 for A.O.R) (Figures 5E,H). In the transgenic *Tg(flt1:tdtomatoe; flt4: yfp)* zebrafish line, an injection of *ephrinb2* MO diminished *flt1*^+^ in the distal SeA and DLAV and reduced *flt4*^+^ in the DLAV and PCV, mimicking phenotypes in the absence of WSS or Notch activity. Transient ectopic overexpression of *ephrinb2* mRNA enhanced the dorsal *flt1*^+^ network in the presence of DAPT, DN-*Notch1b* mRNA, and *Gata1a* MO and further restored regeneration of *flt4*^+^ DLAV and PCV (*n* = 20 per group) (Figures 6A,B). Taken together, our data reveals that tail amputation partitions blood flow to promote the WSS-responsive Notch-ephrinb2 pathway to guide arterialization of a new vascular loop.

## Discussion

Shear stress on vascular walls is known to impart mechanical cues during vascular morphogenesis (41) and angiogenic sprouting following injury (42, 43). Although injury removes blood flow carrying vessels and reduces overall blood supply to the injured tissue, the biomechanical mechanisms underlying WSS-mediated vascular remodeling that guides vascular proliferation and differentiation remain undefined. In this study, using a zebrafish model of vascular injury, we reveal that altering the partitioning of blood flow among SeAs following the amputation of the ECL loop produces a local increase in WSS. We demonstrate that local arterial remodeling leads to augmented WSS in the neighboring SeA. Furthermore, the augmented WSS serves as a key biomechanical cue to upregulate endothelial Notch-ephrinb2 signaling that is needed to drive arterial network in the DLAV and formation of a new loop between DLAV and PCV. Mathematical modeling (Figure 1) clarified that peak WSS can increase even when average WSS, which is constrained by the pressure differences in the network, remains constant.

How does tail amputation increase WSS in the proximal SeA? Average WSS in any vessel is constrained by the pressure drop across the vessel, which increases by 57% following amputation of the ECL. However, we found that a much stronger Notch activity is presented by the unsteadiness of the WSS. In the smallest vessels, passage of a *ds-red*^+^ exerts much larger WSS than plasma, and the peak stress within a vessel is strongly linked to the flux of *ds-red*^+^ (Figure 1). Due to the Zweifach-Fung effect, micro-vessels bifurcate, *ds-red*^+^, and plasma fluxes divide in different ratios. Particularly, *ds-red*^+^ are more likely to enter the larger radius branch of the bifurcation than would be dictated by ratio of flows (44). Prior to amputation, this effect means that SeAs carry lower viscosity than the DA. But after tail amputation, the *ds-red*^+^ reaching the distal DA must drain through one of the SeAs, causing a large increase in *ds-red*^+^ flux and therefore causing WSS.

Before amputation, *ds-red*^+^ drain directly into the PCV through the ECL. Following amputation, the large increase in peak WSS requires that *ds-red*^+^ drain directly into the PCV through SeAs. This ECL emerges very early in embryogenesis (45), but direct anastomoses between arteries and veins are common in microvascular networks and can form even when fine vessels already connect the two, such as the basal artery in the zebrafish midbrain which drains into the DLAV (45). Our results suggest that these loops enable large increases in flow in the proximal vessels following amputation, making them directly responsible for initiating WSS-triggered vessel repair and regeneration. Linking vascular regeneration to WSS is particularly relevant to tissues, such as the embryonic zebrafish trunk, in which oxygen levels are too high to trigger hypoxic vessel regrowth (46). At the same time, the existence of such loops carries physical costs since the oxygenated *ds-red*^+^ and glucose carried in the ECL are transported through the trunk without perfusing the trunk tissues.

Our findings demonstrate that tail amputation promotes partitioning of blood flow to engender WSS-activated Notch activity as an essential stimulus for microvessel regrowth and arteriogenesis (Figure 4, Supplementary Figures 8, 9). The mechano-sensitive Notch signaling pathway is widely recognized to coordinate vascular proliferation and differentiation (47–50). Deletion of Notch1 or Notch4 results in impaired micro-vascular network, whereas EC-specific single allele deletion or pharmacologic inhibition of Delta-like ligand 4 (Dll4) regulates neovascularization (47, 51, 52). Notch signaling is involved in biomechanical cell fate decisions (51) including developing vessels and trans-differentiation of arteries into veins,. In addition, Notch activity guides the sizes of arteries and veins (53). Blood flow-induced endothelial Notch activity systematically remodels the arterial segmental network in developing zebrafish embryos (54) and contributes to vascular identity during caudal fin regeneration (10, 40).

**Figure 7:**
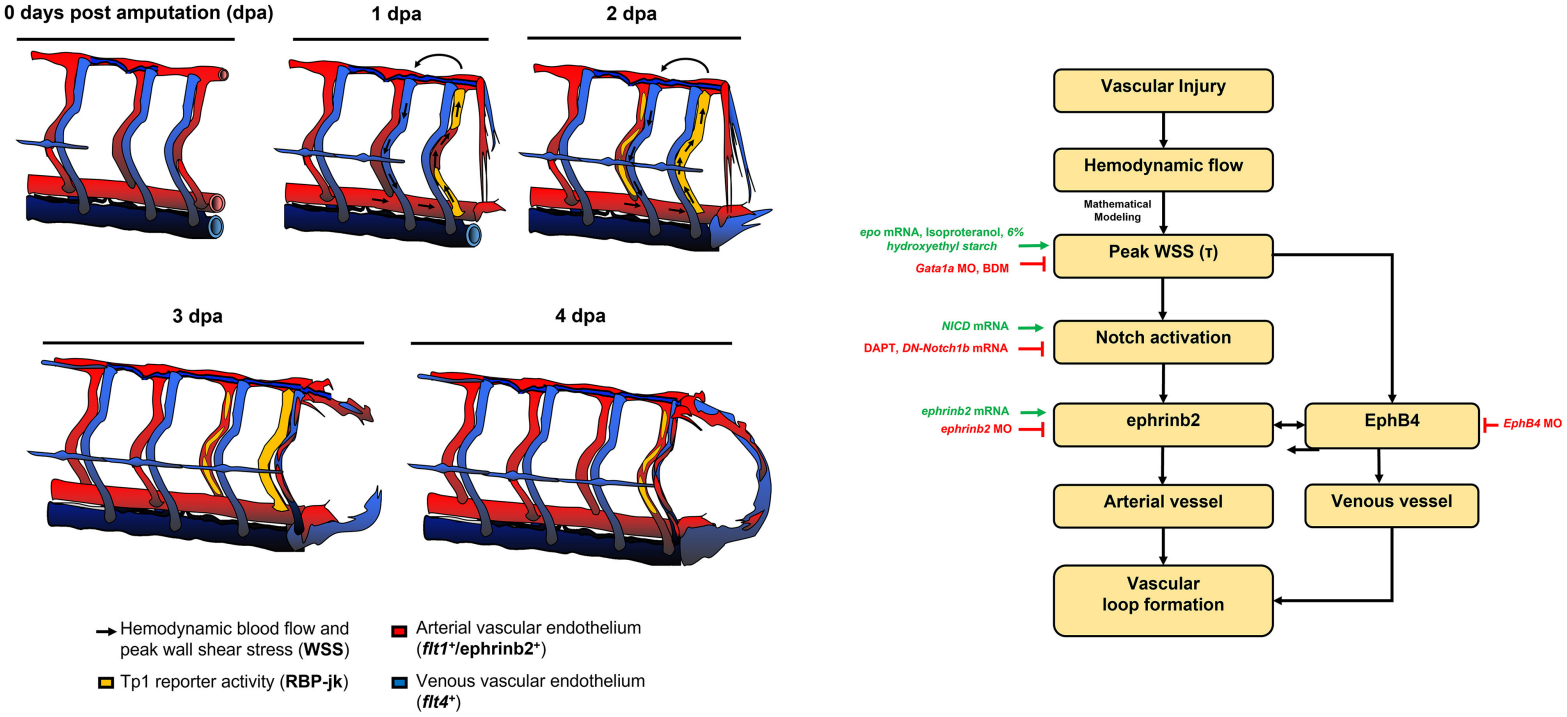
Schematic overviews of the proposed mechanisms underlying WSS-activated regrowth of the vascular loop.

Endothelial ephrinb2 influences angiogenic sprouting and vessel migration (55–58). Ephrinb2 further regulates internalization and subsequent signaling activities of vascular endothelial growth factor receptors (VEGFR2 and VEGFR3). In addition, its expression is prominent at sites of neovascularization or pathological angiogenesis (59, 60). In response to fluid shear stress, reciprocal expression of ephrinb2/EphB4 determines vascular identity (61–63). We observed that endothelial ephrinb2 staining increased in the amputated region (Supplementary Figure 10). Genetic and pharmacological modulations of hemodynamic WSS further supported the notion that augmented WSS is necessary to promote ephrinb2 expression and that ephrinb2 plays a role in establishing the new vascular loop. Our data indicate that endogenous ephrinb2 is a primary target underlying the Notch-dependent arterial network in response to tail amputation (Figure 6). Ephrinb2 is known to regulate postnatal venous neovascularization by modulating EphB4 expression, while ephrinb2 and EphB4 physically interact in growing vessels. These interactions are implicated in cellular intermingling and vascular anastomosis (57, 59, 64, 65). Following tail amputation, we observed that ephrinb2 and EphB4 expressions potentially exhibit synergistic effects to drive vascular loop formation (Supplementary Figures 12A,B). *In vitro* assays showed that the expressions of both ephrinb2 and EphB4 are necessary for endothelial migration and angiogenic tube formation (Supplementary Figures 12C–H). Under hydrodynamic PSS, Notch-dependent ephrinb2 and EphB4 protein expression were upregulated in a time-dependent manner, and the total level of ephrinb2/EphB4 interaction was increased without influencing the polarization kinetics and distribution of the ephrinb2/EphB4 proximity ligations (Supplementary Figures 11C,D). Thus, our observations suggest that amputation-augmented WSS drives arterial Notch-ephrinb2 signaling to regulate lateral venous plexus where EphB4 promotes vessel regeneration. Our proposed mechanism is summarized in Figure 7. In contrast to our results which emphasize reconnections from the arterial network, Xu et al. reported that vein derived arterial cells reconnect vessels following caudal fin regeneration (66). The topology of the trunk network causes largest peak WSS increases in Se arteries, and the dominance of arterial regeneration may reflect localization of WSS signals. Whether reconnection begins with the arterial or venous networks may also depend upon the age- or injury- specific conditions.

Our data connect WSS to vascular loop formation through Notch activated ephrinb2/EphB4 expression. However, both WSS and Notch activation are linked to angiogenic cell proliferation and migration through pathways that may play parallel roles. For example, flow-sensitive microRNAs (miRs), miR-125 and−210, promote tip cell formation and arteriolar branching (67) in response to ischemic injury or hyperlipidemic stress (68, 69). Clusters of miR-497~195 or−449 are implicated in angio- and multicilio-genesis via Notch activation (70, 71). miR-17 and homologs of miR-20 are associated with endothelial ephrinb2 expression (72). In addition, both WSS and Notch activation are connected to metabolic changes. WSS affects endothelial VEGFR2- protein kinase C isoform epsilon signaling to increase the glycolytic metabolite, dihydroxyacetone (26). The Notch co-activator, forkhead box subfamily O1 transcription factor, couples vascular growth with metabolic activity, while Notch signaling triggers the phosphatidylinositol 3-kinase/AKT serine/threonine kinase pathway and regulates glycolysis-related genes during tumor angiogenesis (73, 74). Pharmacologic inhibition of γ-secretase has systemic and irreversible mechanism-of-action to control Notch activity (75), and the selectivity may not be restricted only to the EC population. In addition, a plethora of oncogenetic research suggested other potential effects of DAPT treatment related to caspase-mediated apoptosis and toxicity as a consequence of stress-related gene activation (75, 76).

Lastly, we demonstrated that following vessel injury, the zebrafish trunk vasculature remodels to create a direct anastomotic connection between arteries and veins to focus flow into a single Se vessel. Arteriovenous anastomoses (“shunts”) can be observed across systems and organs (77). Yet, their existence is difficult to explain on the basis of perfusion since they offer blood cells a high conductance route through tissues that circumvents the capillary bed. Anastomoses have been speculated to play a role in pressure and temperature regulation (77). In the zebrafish trunk, the anastomosis between DA and PCV is directly responsible for the repartitioning of flow following amputation and, thus, for generating the WSS signals that initiate sprouting. Follow-up work should examine whether anastomoses in other systems provide the same function of shaping growth following injury, and whether the presence of anastomoses is correlated with the ability to response to an injury.

## Data Availability Statement

The original contributions presented in the study are included in the article/Supplementary Materials, further inquiries can be directed to the corresponding author/s.

## Ethics Statement

The animal study was reviewed and approved by University of California, Los Angeles (UCLA), under animal welfare assurance number A3196-01.

## Author Contributions

KB and C-CC performed zebrafish studies including micro-injections and confocal imaging. KB, S-SC, SC, MRop, and TH wrote the manuscript. KB, S-SC, and YW performed blood flow imaging and mathematical analyses of peak WSS. MRou and JC performed confocal imaging and CFD analyses. KB, C-CC, and YD performed post image processing and batch analyses. KB, JM, and JA performed *in vitro* cell culture, flow exposure, and molecular biology. KB designed experiments. HC, RO'D, SC, MR, and TH supervised, revised, and supported the study. All authors contributed to the article and approved the submitted version.

## Funding

This study was supported by National Institutes of Health R01HL083015 (TH), R01HL111437 (TH), R01HL129727 (TH), R01HL118650 (TH), I01 BX004356 (TH), 5R01GM126556 (MRou), K99HL148493 (YD), 5T32HL007745-28 (KB) and AHA 18CDA34110338 (YD).

## Conflict of Interest

The authors declare that the research was conducted in the absence of any commercial or financial relationships that could be construed as a potential conflict of interest.

## Publisher's Note

All claims expressed in this article are solely those of the authors and do not necessarily represent those of their affiliated organizations, or those of the publisher, the editors and the reviewers. Any product that may be evaluated in this article, or claim that may be made by its manufacturer, is not guaranteed or endorsed by the publisher.

## Supplementary Material

Supplementary Figure 1 Changes in embryonic circulatory loop (ECL) topology via tail amputation localizes blood flow (*Ds-red*^+^) in the amputated site. Tail amputation severed circulation through the ECL, causing *ds-red*^+^ to be routed through nearby segmental arteries (SeAs) between 1–2 dpa (white arrowheads). Scale bar: 20 μm.

Supplementary Figure 2 Average diameter of segmental vessels in the amputated site. Amputated vascular networks in *Tg(fli1: gfp)* zebrafish embryos were imaged to assess vessel diameter for flow adaptation. Average diameters of the caudal SeAs adjacent to the amputated site modestly increased from 1 dpa and remained dilated during regeneration.

Supplementary Figure 3 Notch signaling regulates vessel lumen formation and endothelial collagen 4 expression during vascular loop formation. **(A)** At 4 dpa, endoluminal blood flow was observed in the DMSO-treated embryos (white arrowheads), whereas blood flow remained localized in the amputated site in response to γ-secretase inhibitor (DAPT) treatment (white arrowheads). Scale bar: 20 μm. **(B)** Endothelial *ColIV* expression (*ColIV*^+^ EC) was prominent in the proximal SeA and regenerated vessels in the DMSO-treated controls, but not in DAPT-treated embryos (green, white arrowheads, *n* = 5 per group). Scale bar: 20 μm.

Supplementary Figure 4 Endothelial Notch signaling regulates endothelial cell migration and tube formation *in vitro*. **(A,B)** Representative images of Matrigel tube formation and human aortic endothelial cell (HAEC) migration with or without DAPT treatment or *siNotch1* transfection (Black arrowheads). **(C)** The density quantification of Notch1 protein expressions following *siScr* or *siNotch1* transfection. TCL: total cell lysates. **(D,E)** DAPT treatment or *siNotch1* transfection reduced both tube length and the number of branch points as compared to DMSO-or *siScr-*transfected controls (^**^*p*< *0.005* vs. DMSO, vs. *siScr, n* = 7 for tube length, *n* = 3 for branch point formation). **(F)** DAPT treatment or *siNotch1* transfection significantly attenuated area of recovery (A.O.R) at 12 h post scratch. (^*^*p*< *0.005*, ^**^*p*< *0.005* vs. DMSO, vs. *siScr, n* = 3) **(G)** 6 h of pulsatile shear stress (PSS) exposure *in vitro* upregulated Notch-related gene expressions including Notch ligands Dll4 and Jag1, and the targets Hes1, Hey1, and Hey2. DAPT treatment mitigated PSS-increased mRNA expressions (^*^*p*< *0.05*, ^**^*p*< *0.005*, ^***^*p*< *0.0005* vs. static, normalized with human actin, *n* = 3).

Supplementary Figure 5 Computational fluid dynamics (CFD) to validate hemodynamic WSS modulation. CFD analyses were performed to validate viscosity- and contractility-mediated hemodynamic WSS. While increase in viscosity (*epo* mRNA, 10–20 pg/nl) or myocardial contractility (isoproterenol, 100 μM) increased averaged hemodynamic WSS at 0 dpa, reduction of viscosity (*Gata1a* MO, 1 mM) or myocardial contractility (BDM, 100 μM) reduced WSS in the distal SeA adjacent to the amputated site (white arrow). SeA: Arterial segmental vessel, SeV: Venous segmental vessel.

Supplementary Figure 6 Analyses of 6% hydroxyethyl hetastarch injection via common cardinal vein (CCV). **(A)** Blood flow in embryos injected with 6% hydroxyethyl hetastarch to modulate plasma viscosity was analyzed by co-injecting with FITC-dextran. Images of injected embryos were taken at 1 hour post injection (hpi). DLAV: Dorsal longitudinal anastomotic vessel. CCV: Common cardinal vein. Scale bar: 50 μm. **(B)** Average heart rate (bpm) remained unchanged at 1 hpi (*n* = 5). **(C)** Assessment of changes in plasma viscosity and flow rate following 6% hydroxyethyl hetastarch injection. For the measurements, see Method. **(D,E)** At 1 hpi, *ds-red*^+^ concentration in the DA was reduced by 5.5%, whereas flow rate of *ds-red*^+^ remained unchanged (*n* = 6 for *ds-red*^+^ concentration, *n* = 4 for flow rate measurement). **(F)** Under the static condition, Notch-related gene expressions in HAEC remained unchanged following 4 and 12 h of 6% hydroxyethyl hetastarch treatment (^**^*p*< *0.005*, ^***^*p*< *0.0005* vs. H_2_O, normalized to human actin, *n* = 3).

Supplementary Figure 7 Notch signaling pathway regulates WSS-mediated loop formation. **(A)** Representative images of endothelial *tp1* activity and vessel regeneration following transient modulations of global Notch expression (DAPT, DN-*Notch1b* and *NICD* mRNA) and WSS (*epo* mRNA, isoproterenol, *Gata1a* MO and BDM). In the presence of DAPT or DN-*Notch1b* mRNA, augmented WSS failed to increase endothelial *tp1* activity in the amputated site (white arrowheads) and did not promote loop formation at 4 dpa (white arrows). *NICD* mRNA injection up-regulated endothelial *tp1* activity and restored *Gata1a* MO-impaired regeneration. Following exposure to BDM, *NICD* mRNA increased endothelial *tp1* activity, but failed to restore regeneration. (^*^*p*< *0.05*, ^**^*p*< *0.005*, vs. *NICD* mRNA+*Gata1a* MO, *n* = 20). Scale bar: 20 μm.

Supplementary Figure 8 Time-lapse imaging of arterial and venous vessels with *Tg(Flt1:tdtomatoe; Flt4: yfp)* zebrafish line. Time-lapse images of arterial (*flt1*^+^, red) and venous (*flt*4^+^, green) vessels with indicated treatments. Scale bar: 20 μm.

Supplementary Figure 9 Notch signaling pathway regulates the WSS-responsive arterial network. In the presence of DAPT or DN-*Notch1b* mRNA, increase in viscosity- and contractility-mediated WSS (*epo* mRNA, isoproterenol, 6% hydroxyethyl hetastarch) resulted in the absence of *flt1*^+^network (white arrowheads) for loop formation (white arrow) at 4 dpa. In addition, *flt4*^+^ DLAV and PCV (^*^ asterisk) were partially attenuated at 4 dpa. Conversely, *NICD* mRNA reversed the effect of *Gata1a* MO (*n* = 20 per group). Scale bar: 20 μm.

Supplementary Figure 10 Flow-responsive Ephrinb2 regulates Notch-dependent DLAV-PCV vascular loop formation. **(A)** Whole mount immunofluorescence staining against ephrinb2 (green). At 3 dpf, *Tg(flk1: mcherry)* embryos exhibited high endogenous ephrinb2 expression in their central nervous system, heart, and neural tube (white arrowheads). Scale bar: 100 μm. **(B)** Representative images of endothelial ephrinb2 staining during DLAV-PCV vascular loop formation (white arrowheads). Scale bar: 20 μm. At 1 dpa, endothelial ephrinb2 staining increased in the caudal DA (white arrowhead). Ephrinb2 staining increased in the distal SeA and in regenerated vessels at 4 dpa. Reduction of viscosity- and contractility-mediated WSS via *Gata1a* MO injection and BDM treatment reduced endothelial ephrinb2 staining, whereas increase in hemodynamic WSS (*epo* mRNA, isoproterenol, 6% hydroxyethyl hetastarch) increased ephrinb2 staining in the distal SeA, DLAV and CVP from 2 to 4 dpa. DAPT treatment, as the positive control, reduced ephrinb2 staining in the distal SeA and regenerated vessel regions (*n* = 5 per group). Scale bar: 20 μm.

Supplementary Figure 11 Pulsatile shear stress (PSS) increases total amount of ephrinb2/EphB4 interaction. HAEC monolayers were subjected to unidirectional PSS (23±7 dyne·cm^−2^ at 1Hz) for 6, 12, 24 h. **(A)** Exposure to PSS increased both ephrinb2 and EphB4 protein expression in a time-dependent manner. While DAPT treatment and *siNotch1* transfection attenuated PSS-increased ephrinb2 expressions, EphB4 expression remained statistically unchanged (^*^*p*< *0.05*, ^**^*p*< *0.005*, ^***^*p*< *0.0005* vs. static, normalized to β-tubulin, *n* = 3 per time point). **(B)** Transcript of Kruppel Like Factor 2 (KLF2) mRNA expressions were assessed as an internal control. KLF2 mRNA increased by ~ 4.0-fold following PSS exposure. (^**^*p*< *0.05*, vs. static, normalized to human actin, *n* = 3 per time point). **(C)** Compared to static controls, 6 h of PSS exposure modulated ephrinb2-mediated EphB4 pull down in Notch-dependent manner (untreated: 59%; *siScr*: 65%, *siNotch1*: 18% reduction, DMSO: 21% and DAPT: 16%, respectively, ^*^*p*< *0.05*, vs. static, *n* = 3 per group). **(D)** PSS further increased the total amount of ephrinb2/EphB4 complex without affecting polarization kinetics between endogenous ephrinb2 and EphB4. The average number of individual ephrinb2/EphB4 ligation and average size of the total ligations per cell were quantified for statistical comparisons (White). Compared to the static control, PSS exposure increased average size of an individual ligation by 2.3-fold, while transfection significantly reduced both the number and the size of ligations and attenuated the effect of PSS (^*^*p*< *0.05* vs. static, *n* = 3 for static controls, *n* = 5 for PSS exposure).

Supplementary Figure 12 Endothelial ephrinb2/Ephb4 pathway systematically regulates vascular loop formation. **(A)** Injection of EphB4 MO (0.5 mM) disrupted caudal vein capillary plexus (CVP) morphology (^*^asterisk) and impaired DLAV-PCV loop formation (white arrowheads). In addition, knockdown of both ephrinb2 and EphB4 (0.25 mM) sustained EphB4-knockdown phenotypic patterns and arrested loop formation (*n* = 20 per group). Scale bar: 20 μm. EphB4 or double knockdown inhibited both *flt1*^+^ in the proximal SeA and *flt4*^+^ DLAV and PCV during loop formation (*n* = 20 per group). **(B)** Schematic representations of ephrinb2/EphB4-mediated *flt1*^+^and *flt4*^+^regeneration during vascular loop formation. Black arrowheads depict regenerated *flt1*^+^/*flt*4^+^ following EphB4 or ephrinb2/EphB4 knockdown. **(C,D)** Representative images of *in vitro* Matrigel tube formation and HAEC migration with and without *siEphB4* transfection and/or co-transfection with *siephrinb2*. **(E)** The density quantification of EphB4 expression following *in vitro siEphB4* transfection. TCL: total cell lysate **(F,G)** *siEphB4* transfection reduced tube length and the number of branch points. Transfection of both *siephrinb2* and *siEphB4* aggravated branch point formation compared to *siScr*-transfected HAEC (^*^*p*< *0.05*, ^**^*p*< *0.005*, ^***^*p*< *0.0005* vs. *siScr, n* = 3). **(H)** *siEphB4* transfection reduced, and transfection of both *siephrinb2* and *siEphB4* further reduced A.O.R by at 24 h post scratch (^**^*p*< *0.005*, ^***^*p*< *0.0005* vs. vs. *siScr, n* = 3).

Supplementary Figure 13 Quantification of vascular loop formation. Area quantifications of the vessel regeneration were performed as described in Method. Entire network of endothelial vasculature on the posterior tail segment (purple) was designated automatically, whereas regenerated vascular loop was derived manually (pink).

Supplementary Video 1 Tail amputation increased hemodynamic flow in the distal segmental arteries (SeA).

Supplementary Video 2 Blood flow adaptation in *flt1*^+^ network following tail amputation.
