# Supplementary figures and images for: Vascular Injury in the Zebrafish Tail Modulates Blood Flow and Peak Wall Shear Stress to Restore Embryonic Circular Network
Source: Front Cardiovasc Med. 2022 Mar 18;9:841101. doi: 10.3389/fcvm.2022.841101 (PMC8971683; doi:10.3389/fcvm.2022.841101)

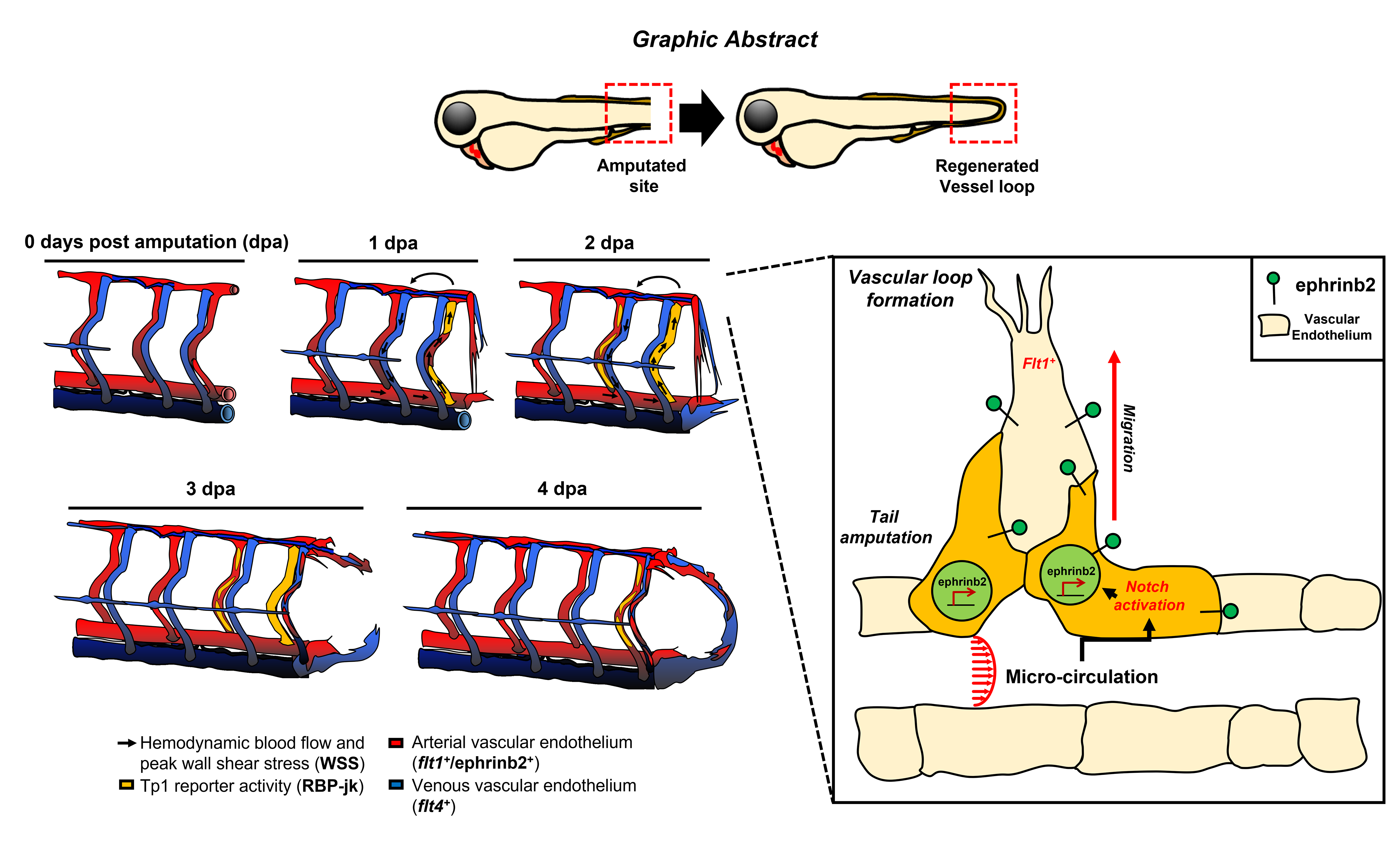

Supplement: Supplementary Figure 1 — Changes in embryonic circulatory loop (ECL) topology via tail amputation localizes blood flow (Ds-red+) in the amputated site. Tail amputation severed circulation through the ECL, causing ds-red+ to be routed through nearby segmental arteries (SeAs) between 1–2 dpa (white arrowheads). Scale bar: 20 μm. [file Image_1.TIF]

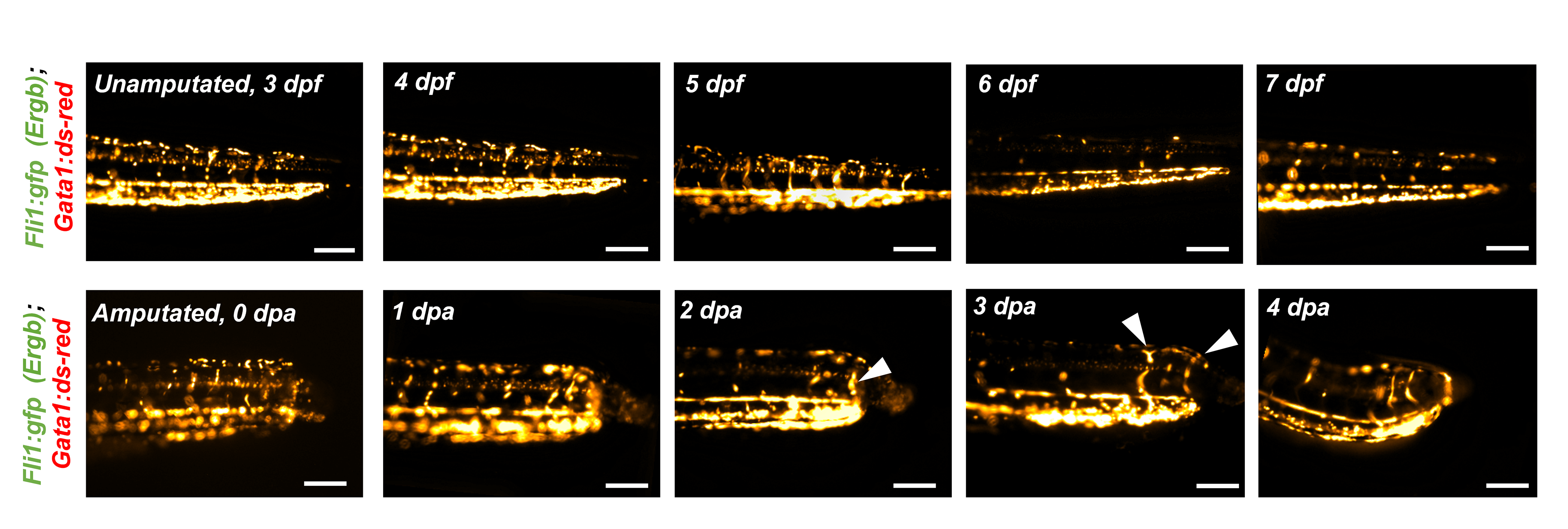

Supplement: Supplementary Figure 2 — Average diameter of segmental vessels in the amputated site. Amputated vascular networks in Tg(fli1: gfp) zebrafish embryos were imaged to assess vessel diameter for flow adaptation. Average diameters of the caudal SeAs adjacent to the amputated site modestly increased from 1 dpa and remained dilated during regeneration. [file Image_2.TIF]

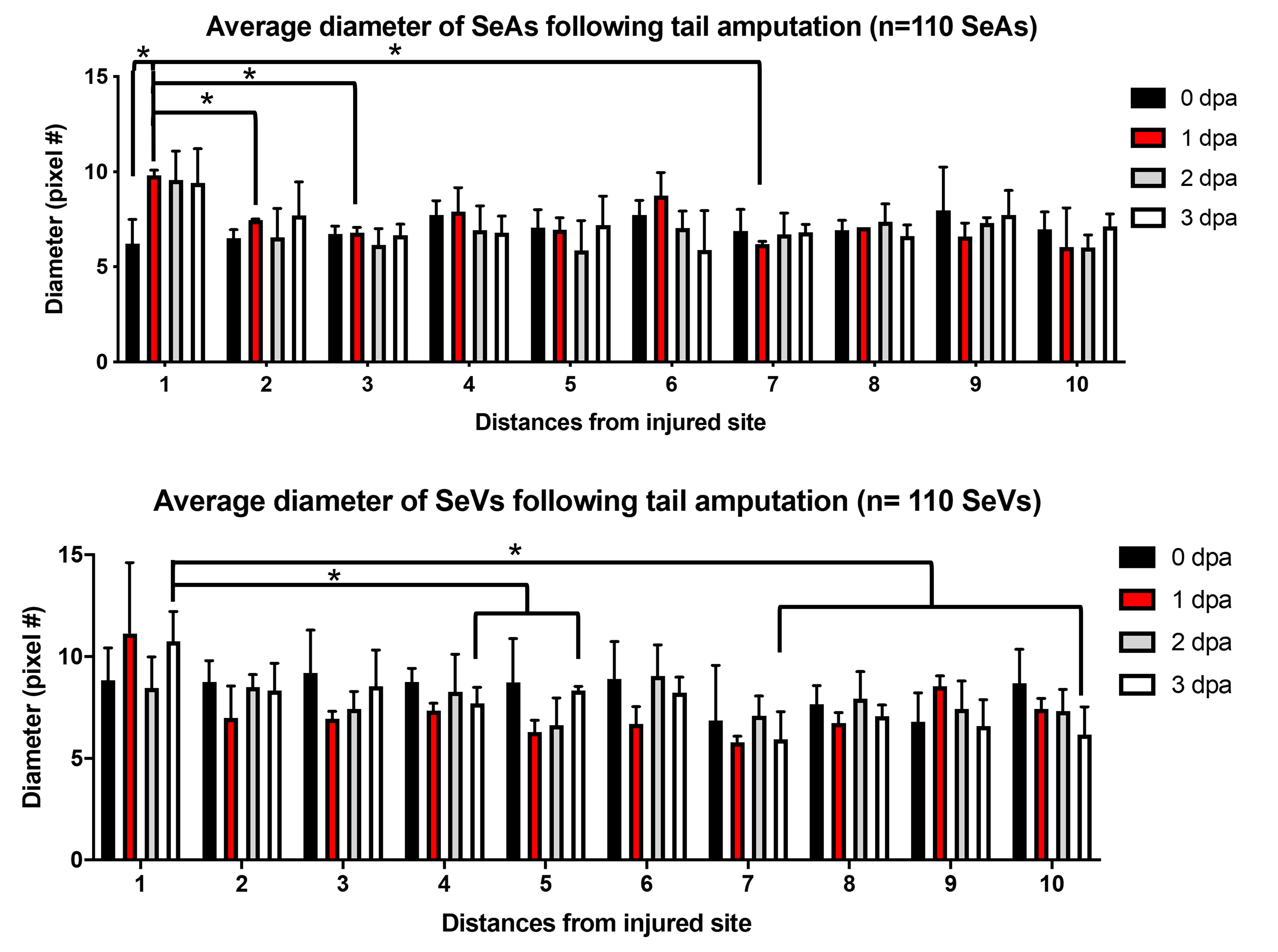

Supplement: Supplementary Figure 3 — Notch signaling regulates vessel lumen formation and endothelial collagen 4 expression during vascular loop formation. (A) At 4 dpa, endoluminal blood flow was observed in the DMSO-treated embryos (white arrowheads), whereas blood flow remained localized in the amputated site in response to γ-secretase inhibitor (DAPT) treatment (white arrowheads). Scale bar: 20 μm. (B) Endothelial ColIV expression (ColIV+ EC) was prominent in the proximal SeA and regenerated vessels in the DMSO-treated controls, but not in DAPT-treated embryos (green, white arrowheads, n = 5 per group). Scale bar: 20 μm. [file Image_3.TIF]

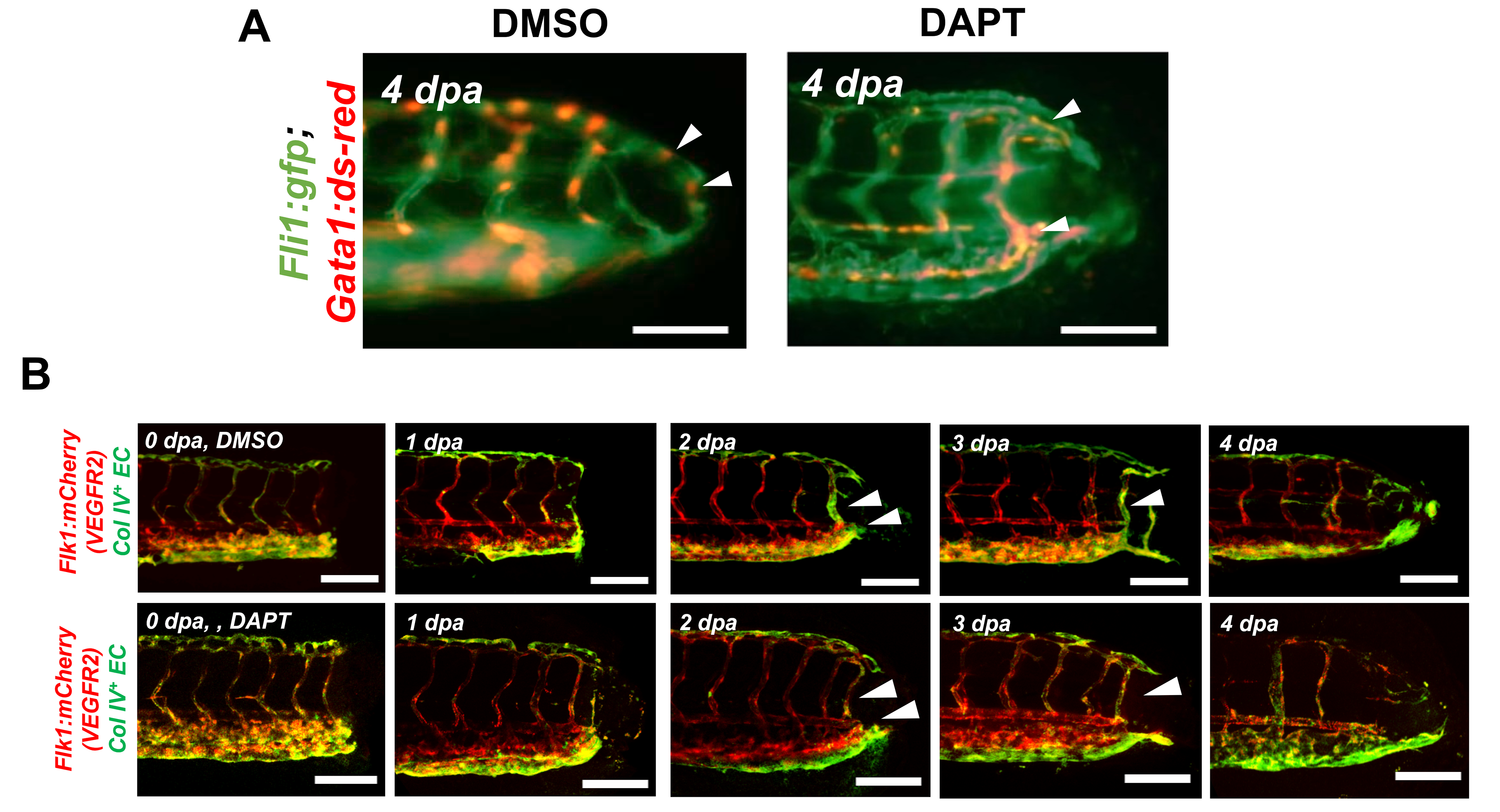

Supplement: Supplementary Figure 4 — Endothelial Notch signaling regulates endothelial cell migration and tube formation in vitro. (A,B) Representative images of Matrigel tube formation and human aortic endothelial cell (HAEC) migration with or without DAPT treatment or siNotch1 transfection (Black arrowheads). (C) The density quantification of Notch1 protein expressions following siScr or siNotch1 transfection. TCL: total cell lysates. (D,E) DAPT treatment or siNotch1 transfection reduced both tube length and the number of branch points as compared to DMSO-or siScr-transfected controls (**p< 0.005 vs. DMSO, vs. siScr, n = 7 for tube length, n = 3 for branch point formation). (F) DAPT treatment or siNotch1 transfection significantly attenuated area of recovery (A.O.R) at 12 h post scratch. (*p< 0.005, **p< 0.005 vs. DMSO, vs. siScr, n = 3) (G) 6 h of pulsatile shear stress (PSS) exposure in vitro upregulated Notch-related gene expressions including Notch ligands Dll4 and Jag1, and the targets Hes1, Hey1, and Hey2. DAPT treatment mitigated PSS-increased mRNA expressions (*p< 0.05, **p< 0.005, ***p< 0.0005 vs. static, normalized with human actin, n = 3). [file Image_4.TIF]

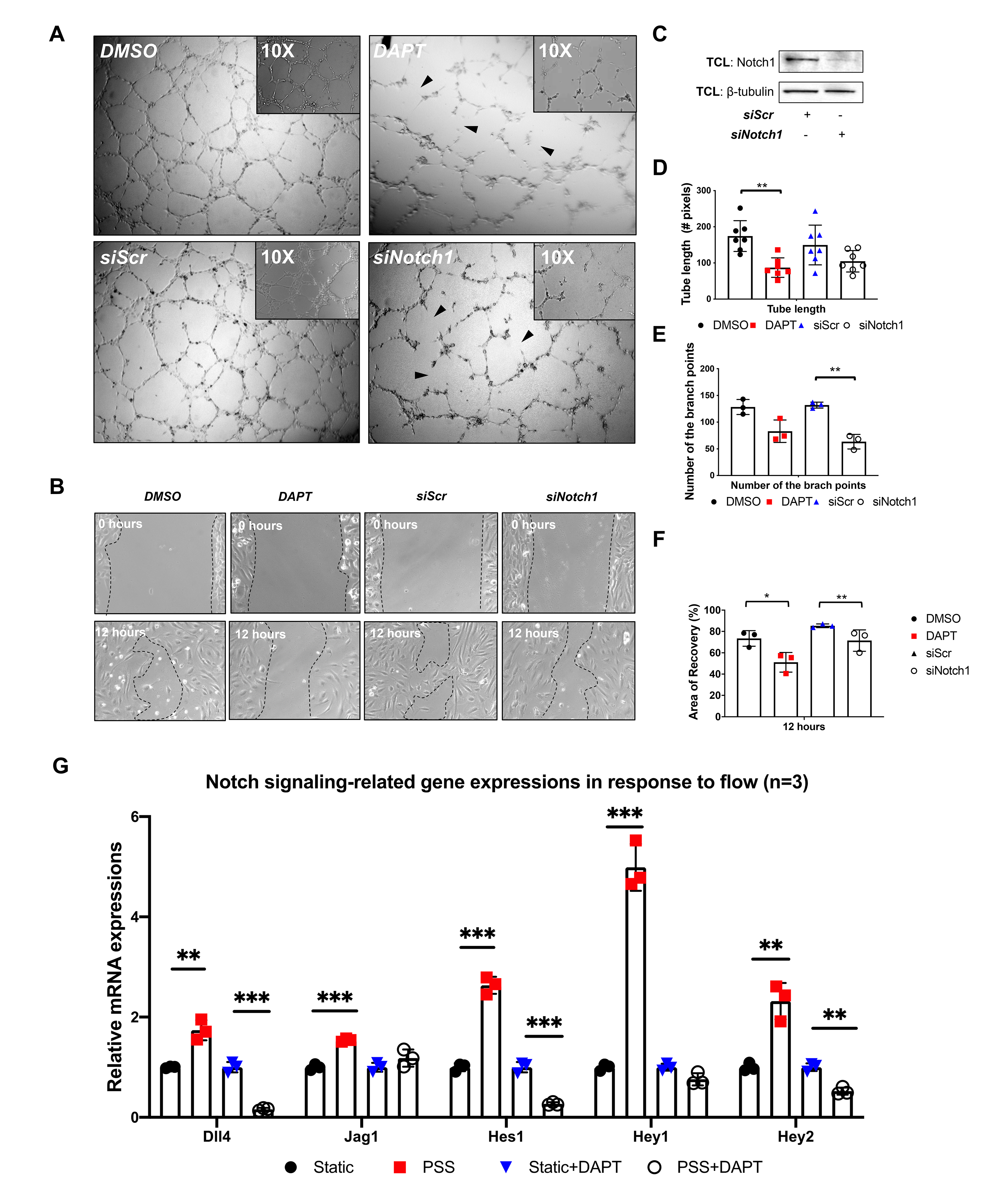

Supplement: Supplementary Figure 5 — Computational fluid dynamics (CFD) to validate hemodynamic WSS modulation. CFD analyses were performed to validate viscosity- and contractility-mediated hemodynamic WSS. While increase in viscosity (epo mRNA, 10–20 pg/nl) or myocardial contractility (isoproterenol, 100 μM) increased averaged hemodynamic WSS at 0 dpa, reduction of viscosity (Gata1a MO, 1 mM) or myocardial contractility (BDM, 100 μM) reduced WSS in the distal SeA adjacent to the amputated site (white arrow). SeA: Arterial segmental vessel, SeV: Venous segmental vessel. [file Image_5.TIF]

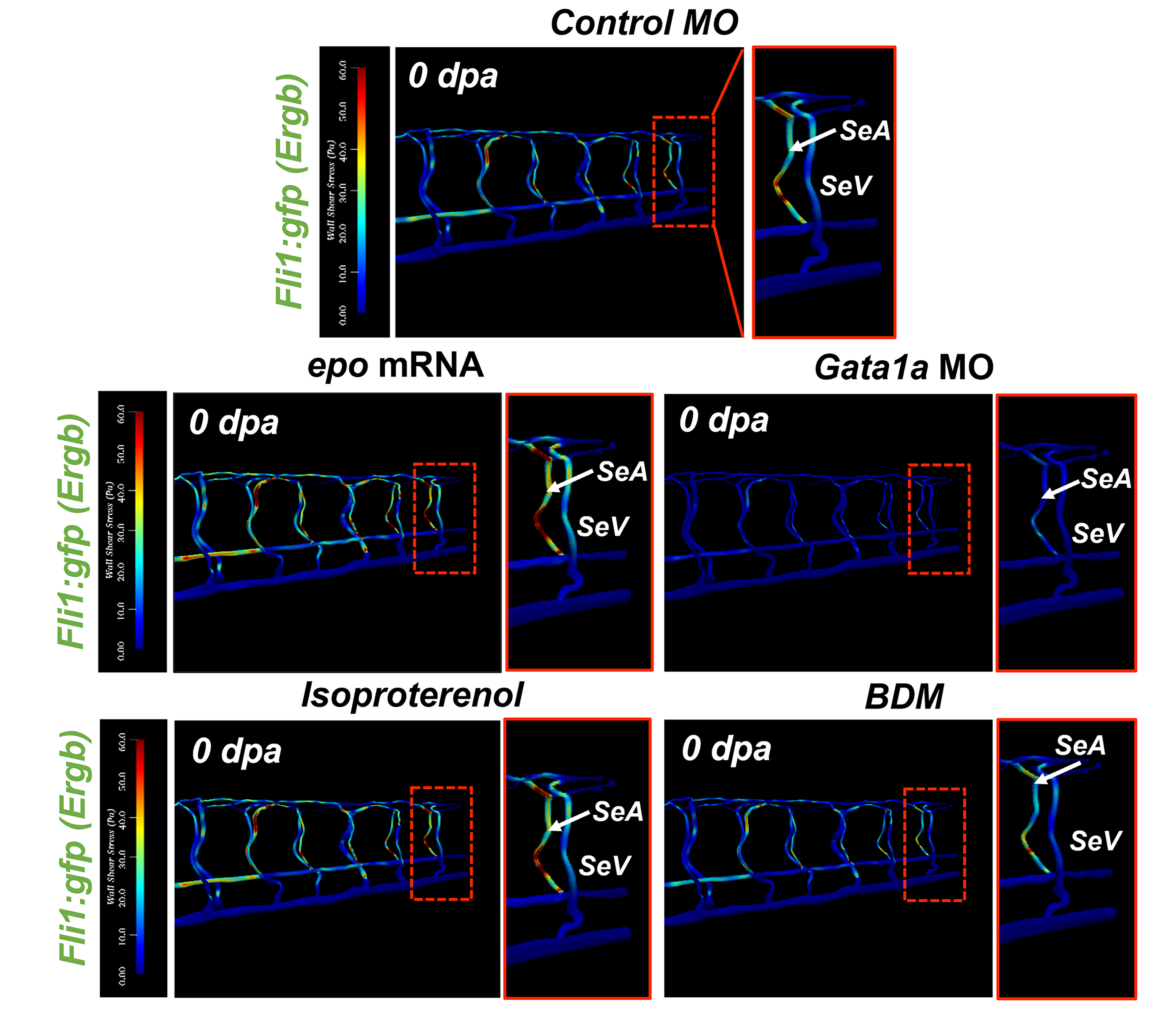

Supplement: Supplementary Figure 6 — Analyses of 6% hydroxyethyl hetastarch injection via common cardinal vein (CCV). (A) Blood flow in embryos injected with 6% hydroxyethyl hetastarch to modulate plasma viscosity was analyzed by co-injecting with FITC-dextran. Images of injected embryos were taken at 1 hour post injection (hpi). DLAV: Dorsal longitudinal anastomotic vessel. CCV: Common cardinal vein. Scale bar: 50 μm. (B) Average heart rate (bpm) remained unchanged at 1 hpi (n = 5). (C) Assessment of changes in plasma viscosity and flow rate following 6% hydroxyethyl hetastarch injection. For the measurements, see Method. (D,E) At 1 hpi, ds-red+ concentration in the DA was reduced by 5.5%, whereas flow rate of ds-red+ remained unchanged (n = 6 for ds-red+ concentration, n = 4 for flow rate measurement). (F) Under the static condition, Notch-related gene expressions in HAEC remained unchanged following 4 and 12 h of 6% hydroxyethyl hetastarch treatment (**p< 0.005, ***p< 0.0005 vs. H2O, normalized to human actin, n = 3). [file Image_6.TIF]

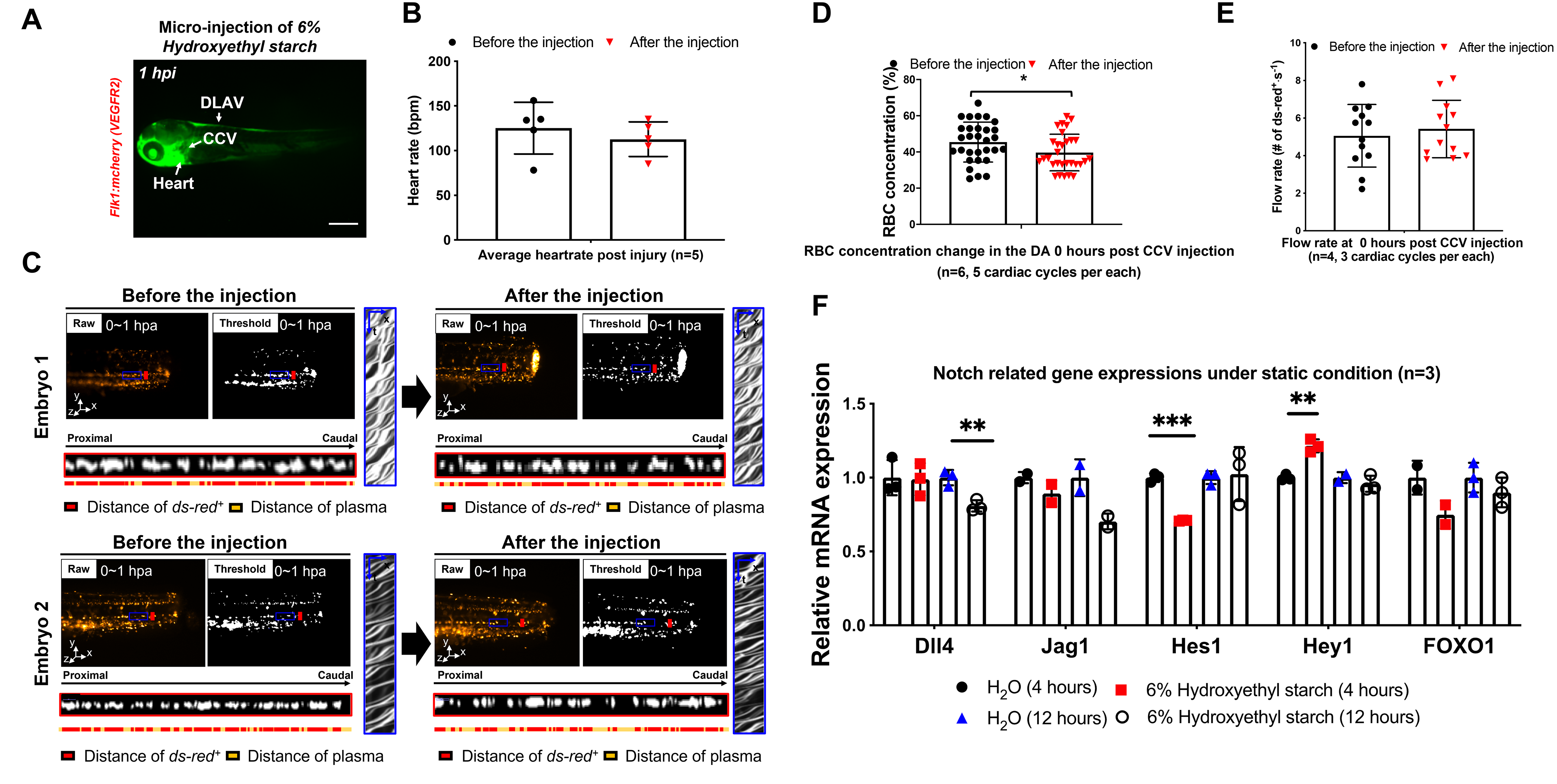

Supplement: Supplementary Figure 7 — Notch signaling pathway regulates WSS-mediated loop formation. (A) Representative images of endothelial tp1 activity and vessel regeneration following transient modulations of global Notch expression (DAPT, DN-Notch1b and NICD mRNA) and WSS (epo mRNA, isoproterenol, Gata1a MO and BDM). In the presence of DAPT or DN-Notch1b mRNA, augmented WSS failed to increase endothelial tp1 activity in the amputated site (white arrowheads) and did not promote loop formation at 4 dpa (white arrows). NICD mRNA injection up-regulated endothelial tp1 activity and restored Gata1a MO-impaired regeneration. Following exposure to BDM, NICD mRNA increased endothelial tp1 activity, but failed to restore regeneration. (*p< 0.05, **p< 0.005, vs. NICD mRNA+Gata1a MO, n = 20). Scale bar: 20 μm. [file Image_7.TIF]

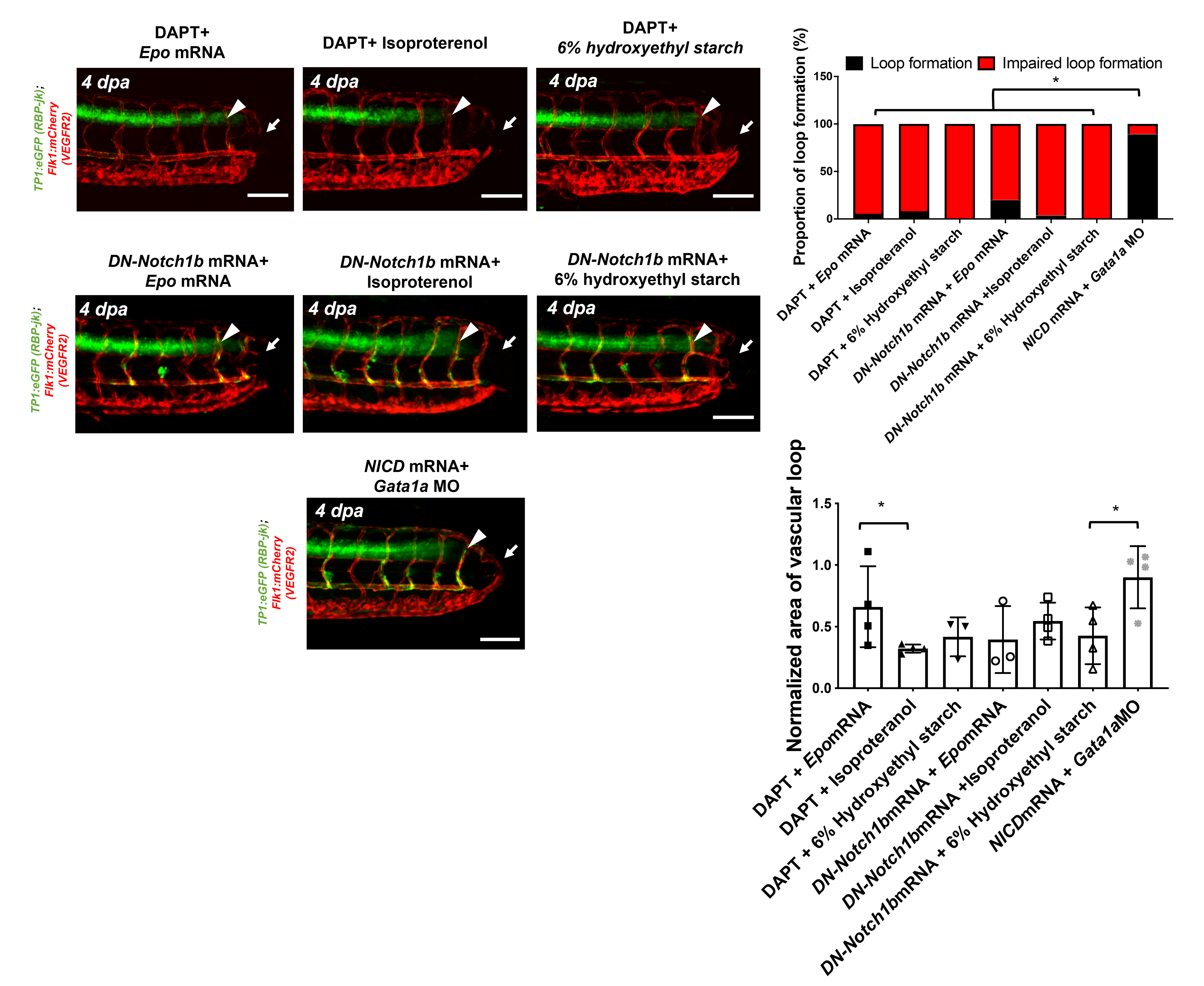

Supplement: Supplementary Figure 8 — Time-lapse imaging of arterial and venous vessels with Tg(Flt1:tdtomatoe; Flt4: yfp) zebrafish line. Time-lapse images of arterial (flt1+, red) and venous (flt4+, green) vessels with indicated treatments. Scale bar: 20 μm. [file Image_8.TIF]

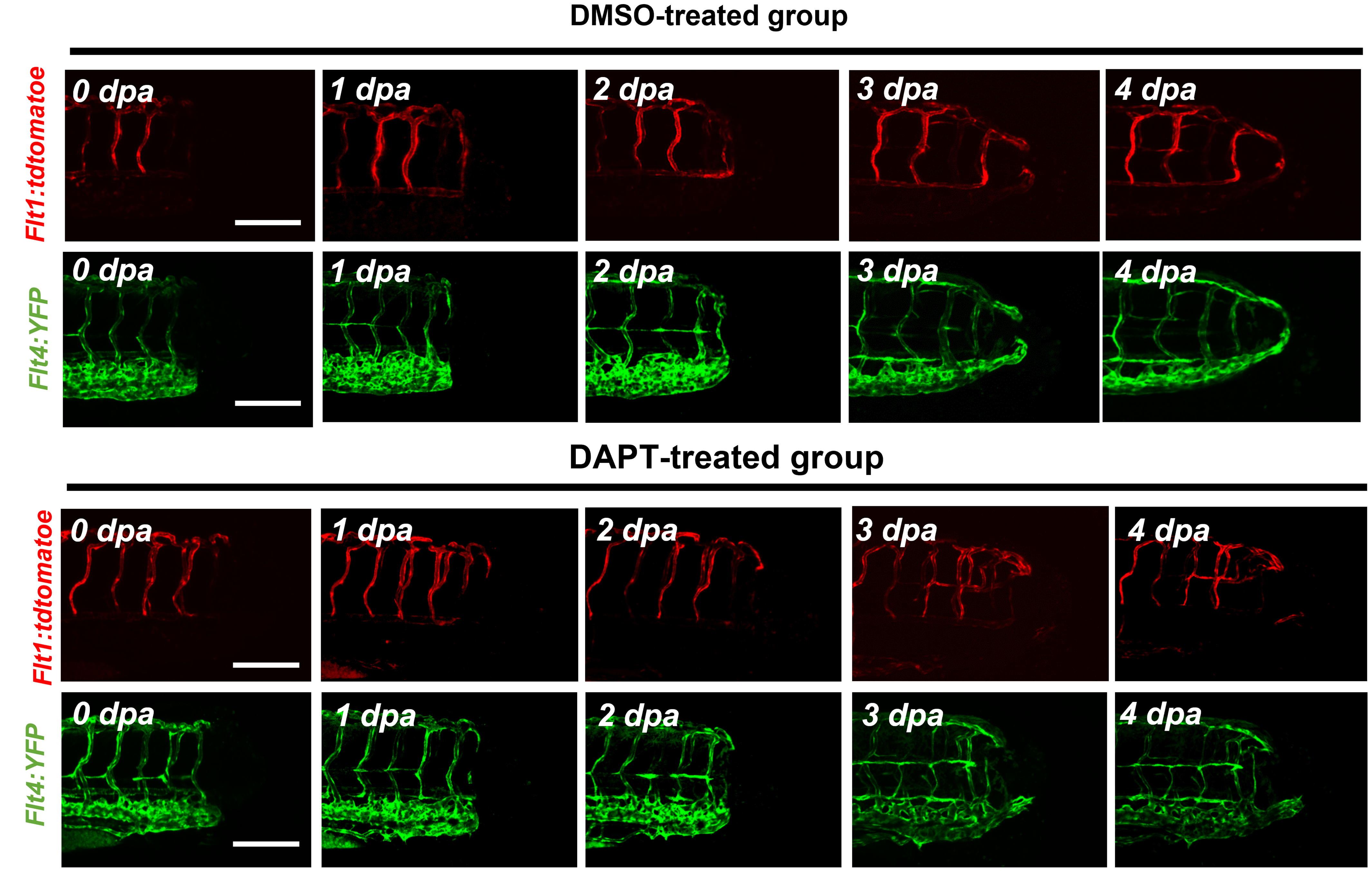

Supplement: Supplementary Figure 9 — Notch signaling pathway regulates the WSS-responsive arterial network. In the presence of DAPT or DN-Notch1b mRNA, increase in viscosity- and contractility-mediated WSS (epo mRNA, isoproterenol, 6% hydroxyethyl hetastarch) resulted in the absence of flt1+network (white arrowheads) for loop formation (white arrow) at 4 dpa. In addition, flt4+ DLAV and PCV (* asterisk) were partially attenuated at 4 dpa. Conversely, NICD mRNA reversed the effect of Gata1a MO (n = 20 per group). Scale bar: 20 μm. [file Image_9.TIF]

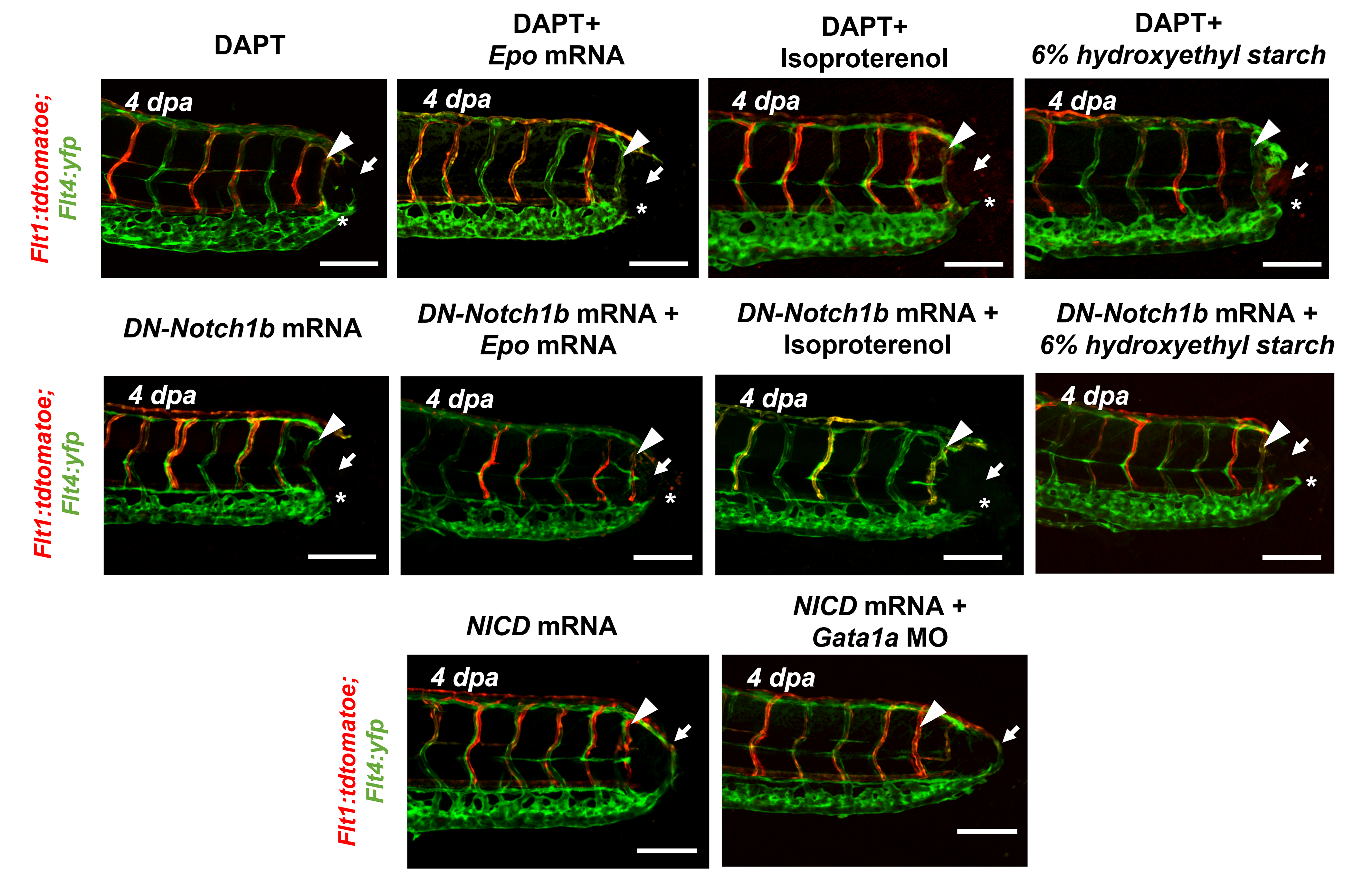

Supplement: Supplementary Figure 10 — Flow-responsive Ephrinb2 regulates Notch-dependent DLAV-PCV vascular loop formation. (A) Whole mount immunofluorescence staining against ephrinb2 (green). At 3 dpf, Tg(flk1: mcherry) embryos exhibited high endogenous ephrinb2 expression in their central nervous system, heart, and neural tube (white arrowheads). Scale bar: 100 μm. (B) Representative images of endothelial ephrinb2 staining during DLAV-PCV vascular loop formation (white arrowheads). Scale bar: 20 μm. At 1 dpa, endothelial ephrinb2 staining increased in the caudal DA (white arrowhead). Ephrinb2 staining increased in the distal SeA and in regenerated vessels at 4 dpa. Reduction of viscosity- and contractility-mediated WSS via Gata1a MO injection and BDM treatment reduced endothelial ephrinb2 staining, whereas increase in hemodynamic WSS (epo mRNA, isoproterenol, 6% hydroxyethyl hetastarch) increased ephrinb2 staining in the distal SeA, DLAV and CVP from 2 to 4 dpa. DAPT treatment, as the positive control, reduced ephrinb2 staining in the distal SeA and regenerated vessel regions (n = 5 per group). Scale bar: 20 μm. [file Image_10.TIF]

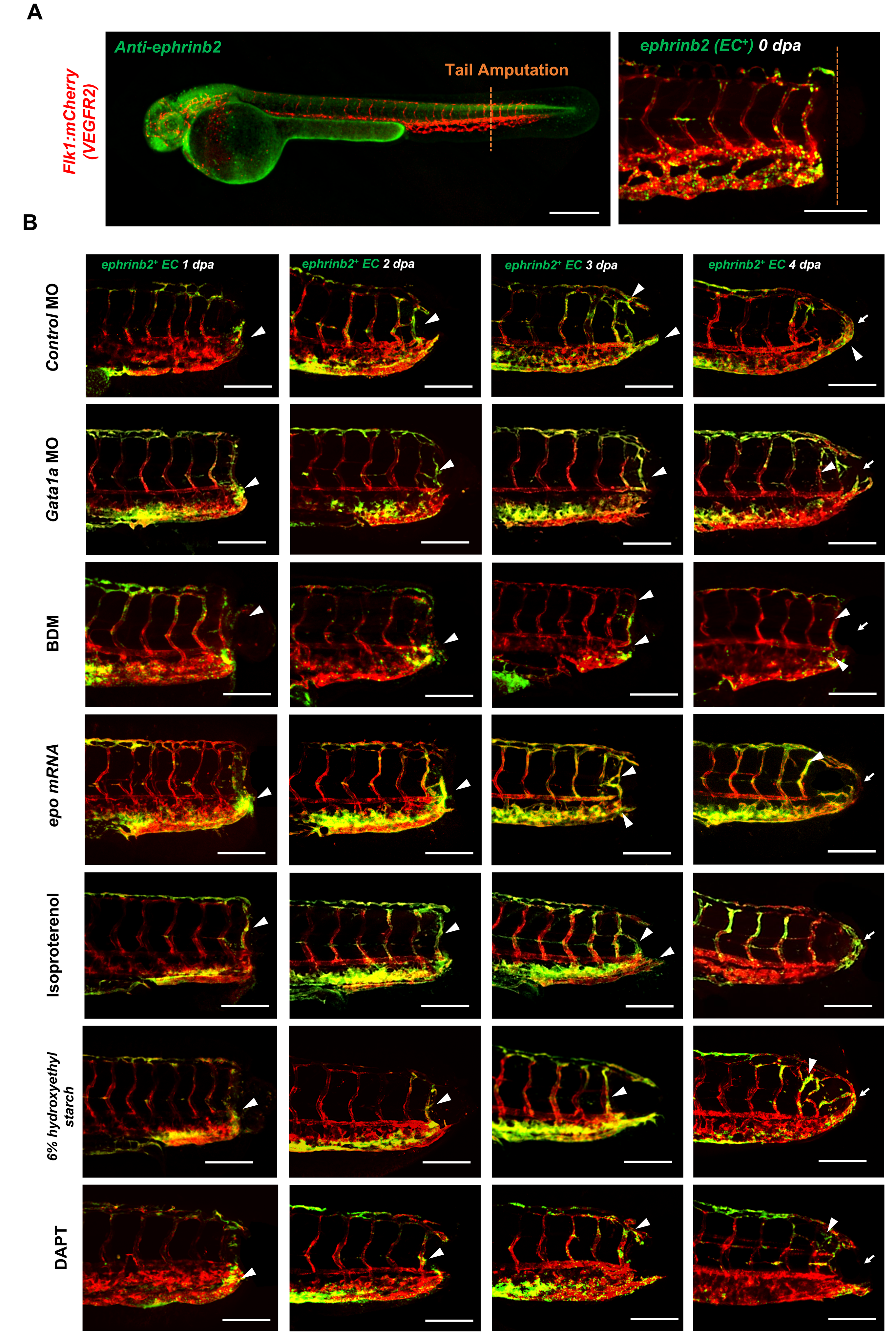

Supplement: Supplementary Figure 11 — Pulsatile shear stress (PSS) increases total amount of ephrinb2/EphB4 interaction. HAEC monolayers were subjected to unidirectional PSS (23±7 dyne·cm−2 at 1Hz) for 6, 12, 24 h. (A) Exposure to PSS increased both ephrinb2 and EphB4 protein expression in a time-dependent manner. While DAPT treatment and siNotch1 transfection attenuated PSS-increased ephrinb2 expressions, EphB4 expression remained statistically unchanged (*p< 0.05, **p< 0.005, ***p< 0.0005 vs. static, normalized to β-tubulin, n = 3 per time point). (B) Transcript of Kruppel Like Factor 2 (KLF2) mRNA expressions were assessed as an internal control. KLF2 mRNA increased by ~ 4.0-fold following PSS exposure. (**p< 0.05, vs. static, normalized to human actin, n = 3 per time point). (C) Compared to static controls, 6 h of PSS exposure modulated ephrinb2-mediated EphB4 pull down in Notch-dependent manner (untreated: 59%; siScr: 65%, siNotch1: 18% reduction, DMSO: 21% and DAPT: 16%, respectively, *p< 0.05, vs. static, n = 3 per group). (D) PSS further increased the total amount of ephrinb2/EphB4 complex without affecting polarization kinetics between endogenous ephrinb2 and EphB4. The average number of individual ephrinb2/EphB4 ligation and average size of the total ligations per cell were quantified for statistical comparisons (White). Compared to the static control, PSS exposure increased average size of an individual ligation by 2.3-fold, while transfection significantly reduced both the number and the size of ligations and attenuated the effect of PSS (*p< 0.05 vs. static, n = 3 for static controls, n = 5 for PSS exposure). [file Image_11.TIF]

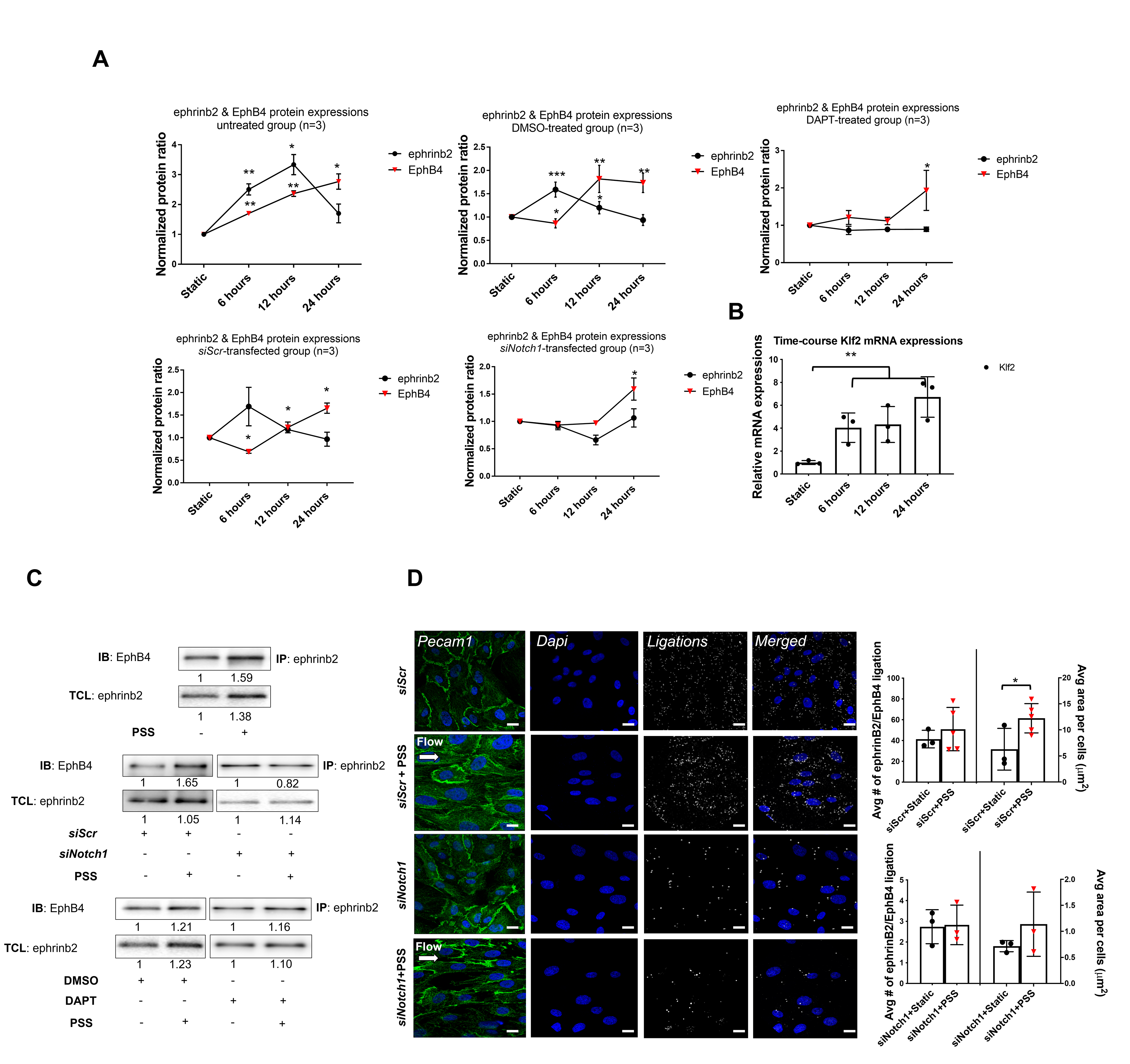

Supplement: Supplementary Figure 12 — Endothelial ephrinb2/Ephb4 pathway systematically regulates vascular loop formation. (A) Injection of EphB4 MO (0.5 mM) disrupted caudal vein capillary plexus (CVP) morphology (*asterisk) and impaired DLAV-PCV loop formation (white arrowheads). In addition, knockdown of both ephrinb2 and EphB4 (0.25 mM) sustained EphB4-knockdown phenotypic patterns and arrested loop formation (n = 20 per group). Scale bar: 20 μm. EphB4 or double knockdown inhibited both flt1+ in the proximal SeA and flt4+ DLAV and PCV during loop formation (n = 20 per group). (B) Schematic representations of ephrinb2/EphB4-mediated flt1+and flt4+regeneration during vascular loop formation. Black arrowheads depict regenerated flt1+/flt4+ following EphB4 or ephrinb2/EphB4 knockdown. (C,D) Representative images of in vitro Matrigel tube formation and HAEC migration with and without siEphB4 transfection and/or co-transfection with siephrinb2. (E) The density quantification of EphB4 expression following in vitro siEphB4 transfection. TCL: total cell lysate (F,G) siEphB4 transfection reduced tube length and the number of branch points. Transfection of both siephrinb2 and siEphB4 aggravated branch point formation compared to siScr-transfected HAEC (*p< 0.05, **p< 0.005, ***p< 0.0005 vs. siScr, n = 3). (H) siEphB4 transfection reduced, and transfection of both siephrinb2 and siEphB4 further reduced A.O.R by at 24 h post scratch (**p< 0.005, ***p< 0.0005 vs. vs. siScr, n = 3). [file Image_12.TIF]

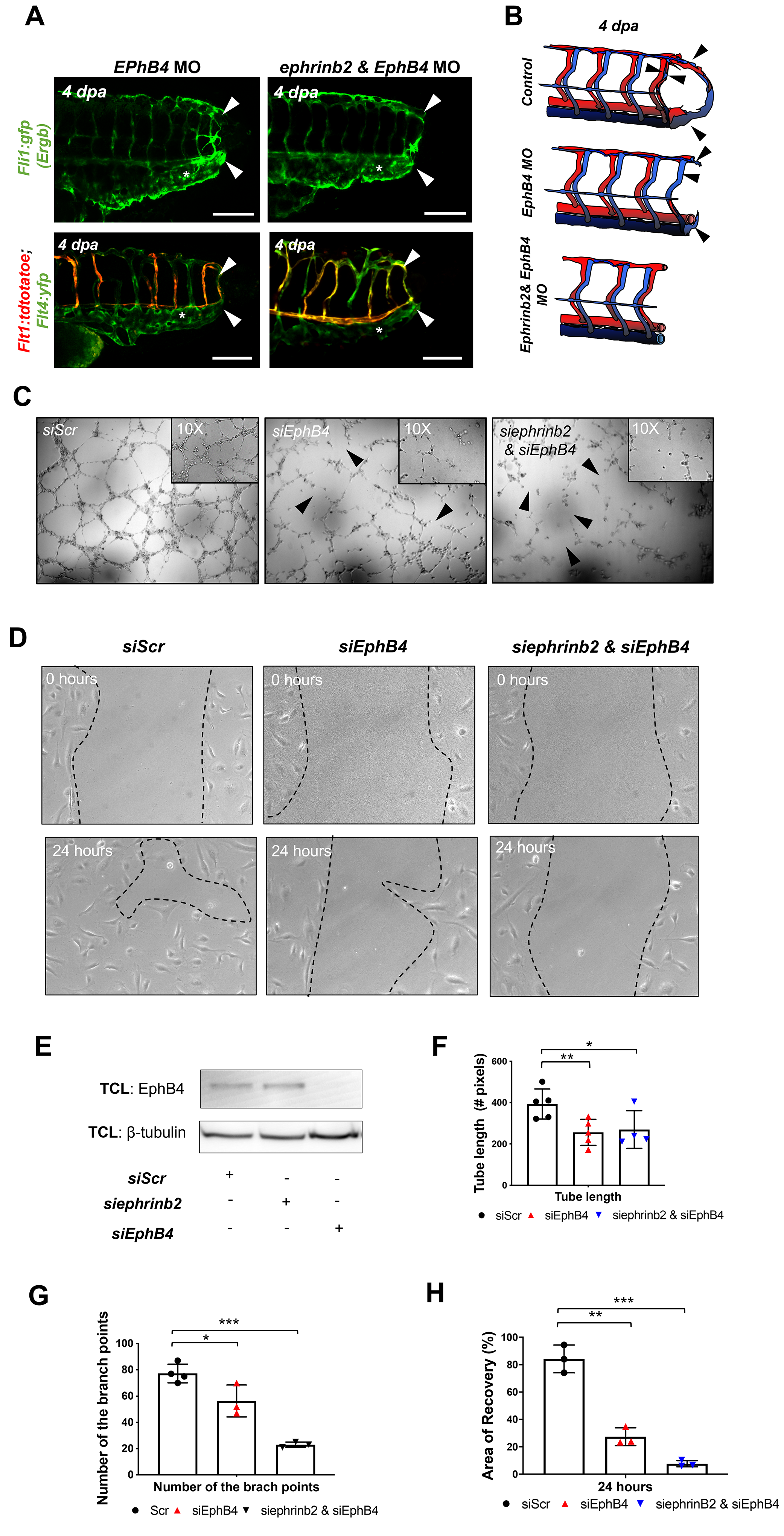

Supplement: Supplementary Figure 13 — Quantification of vascular loop formation. Area quantifications of the vessel regeneration were performed as described in Method. Entire network of endothelial vasculature on the posterior tail segment (purple) was designated automatically, whereas regenerated vascular loop was derived manually (pink). [file Image_13.TIF]

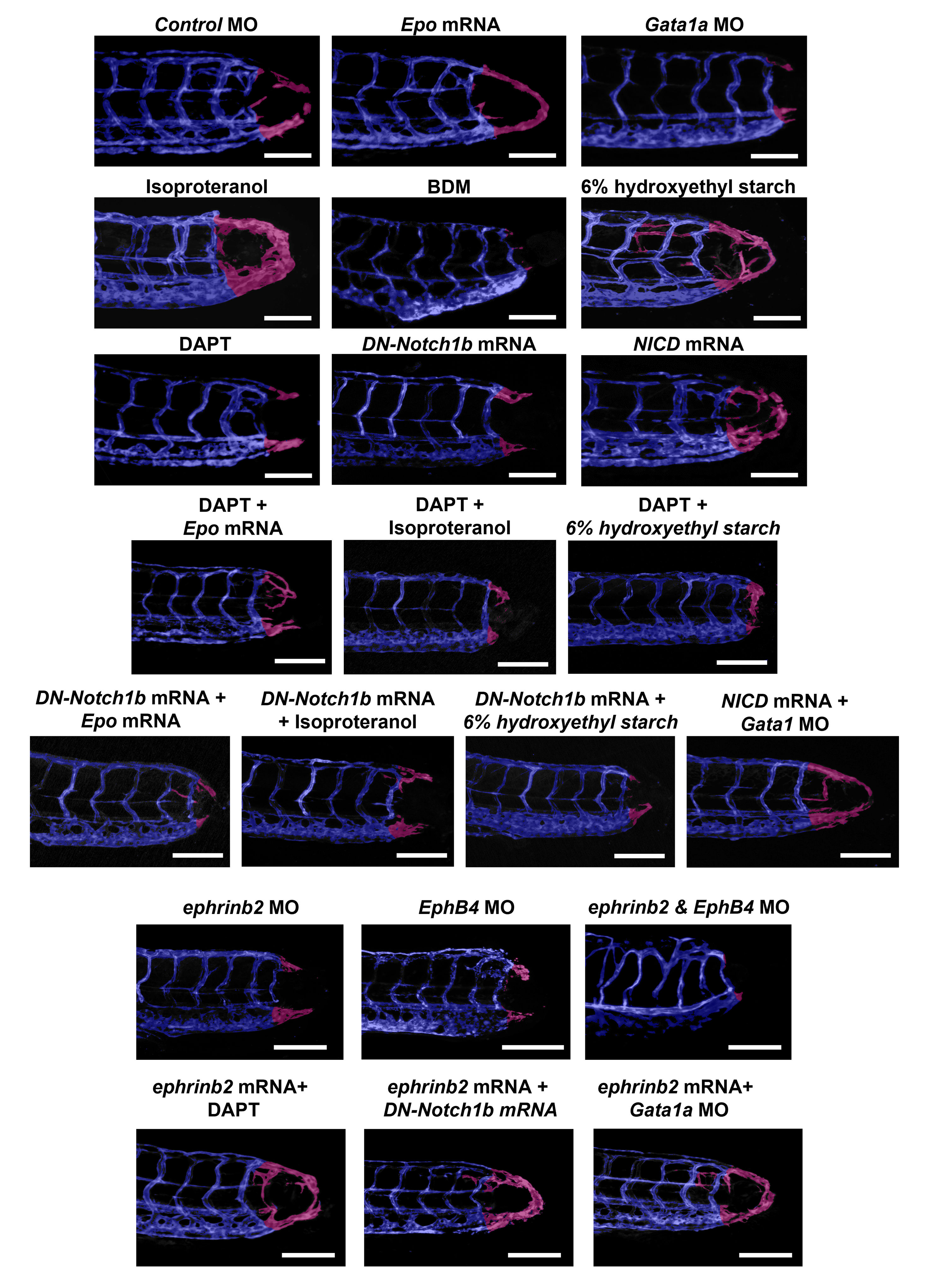

Supplement: Supplementary file 19 [file Image_14.TIF]
